# Entropic and Geometric Population–Coherence Complementarity in Finite-Dimensional Quantum States

**DOI:** 10.3390/e28080877

**Published:** 2026-08-04

**Authors:** José J. Gil

**Affiliations:** Department of Applied Physics, University of Cantabria, Av. Los Castros 48, 39005 Santander, Spain; ppgil@unizar.es

**Keywords:** density matrix, quantum information, intrinsic reference basis, metaspin tensor, population–coherence complementarity, Rényi entropy, majorization, imaginarity, state-space geometry, resource theory, 15A18, 15A63, 81P16, 94A17

## Abstract

Finite-dimensional density matrices contain two representation-intrinsic sectors after the real part is diagonalized, namely ordered intrinsic populations and antisymmetric imaginary coherences. This article develops exact complementarity identities showing how these sectors determine purity, spectral concentration, and entropy. Populations are described by indices of population asymmetry, while coherences are described by the Youla spectrum of the dimensionless metaspin tensor and by correlation-asymmetry indices. In the aligned class, where Youla two-planes coincide with pairs of intrinsic axes, normalized purity splits into a population hierarchy and pairwise coherence terms weighted by products of intrinsic populations. For arbitrary orientations, the coherence term is expressed as a positive semi-definite bilinear form in population-weighted Plücker coordinates. For fixed populations and pairing, increasing any Youla value sharpens the spectrum by majorization and decreases all Rényi entropies, including the von Neumann limit. For fixed ordered populations, maximum aligned cohesion is obtained by saturating adjacent population pairs. The dimensional transition of the discriminating-component cohesion bound is then interpreted as the change from one to two simultaneously saturating metaspin pairs.

## 1. Introduction

Finite-dimensional density matrices are the basic objects of quantum-state theory, quantum information, and statistical descriptions of finite-level systems [1,2,3]. Their eigenvalues determine all unitarily invariant spectral quantities, including purity and entropy, but many structurally meaningful features of a state are not visible from the spectrum alone. They depend on how populations and coherences are distributed in a physically or mathematically distinguished representation. This tension between spectral invariance and representation-dependent structure is central to the geometry of mixed states, to resource theories of coherence and imaginarity [4,5,6], and to the analysis of finite-dimensional covariance and density matrices.

From a quantum-information perspective, the motivation is the following. Many finite-level problems are formulated relative to a real structure fixed by the experimental, computational, or symmetry context. Once that structure is fixed, the imaginary part of a density matrix identifies phase-type coherences that disappear under conjugation and interact with diagonal population uncertainty. Standard resource theories usually begin with an externally selected incoherent basis or a specified set of free states [7,8,9]. The aim here is different and narrower. The real structure is fixed, but the orthogonal frame is selected by the state through Reρ. This gives a state-adapted way to separate the contributions of population imbalance and intrinsic imaginary coherence to spectral purity and entropy reduction.

Information-theoretic complementarity relations for quantum coherence were developed by Cheng and Hall [10]. More recently, finite-dimensional complementarity relations have related coherence or imaginarity to mixedness and predictability in externally prescribed bases or fixed real structures. In particular, Machado et al. [11] resolve the distribution of $l_{1}$-norm coherence in high-dimensional complementarity relations, whereas Chen et al. [12] analyze complementarity and distribution of imaginarity under conjugate symmetry. The present work addresses a distinct but neighboring question. After a real structure has been specified, the reference basis is selected by Reρ itself, the antisymmetric content is organized by the Youla spectrum of the metaspin tensor, and complementarity is refined through population-weighted Plücker geometry and entropy-majorization results. This distinction is essential for the novelty and scope of the results below.

The present work develops a representation-intrinsic way of separating and quantifying two complementary sectors of a density matrix. Given a density matrix ρ, one first applies a real orthogonal transformation that diagonalizes its real symmetric part. The resulting intrinsic reference basis (IRB) [13] puts the state in the form

(1) $$\rho_{O}=A+iN,$$

where $A=\mathrm{diag}(a_{1},\ldots ,a_{n})$ contains the ordered intrinsic populations and *N* is a real antisymmetric matrix containing the imaginary coherences in that intrinsic frame. The populations $a_{i}$ are organized by the indices of population asymmetry [14,15]. The antisymmetric sector is organized by the dimensionless metaspin tensor [16], whose Youla canonical form supplies a hierarchy of correlation-asymmetry indices. The two hierarchies are not merely parallel lists of descriptors. Together, they determine how population imbalance and intrinsic imaginary coherence combine to produce spectral sharpening, purity, and entropy.

Throughout this work, the adjective *intrinsic* is relative to a specified real structure on the Hilbert space. Once the conjugation operation used to define Reρ and Imρ is fixed, the IRB is determined by Reρ up to residual real-orthogonal freedom. The descriptors developed here are invariant under real orthogonal changes preserving that real structure, but not under arbitrary complex unitary similarities, which can redistribute real and imaginary matrix content. In quantum-information settings, the construction is therefore applicable whenever the real structure is operationally fixed, for example, by a computational basis, an antiunitary symmetry, or a physically specified real frame.

The first objective of the article is to refine the scalar population–coherence complementarity identity into a hierarchy. In the aligned class, where the principal Youla two-planes of the metaspin tensor coincide with pairs of IRB axes, the normalized purity decomposes into a population term plus pairwise coherence terms. Each coherence contribution is weighted by the product of the two populations in the corresponding intrinsic pair. This yields a level-resolved complementarity identity that distinguishes states with the same scalar purity but different distributions of imaginary coherence across intrinsic two-level sectors. In arbitrary orientation, the same contribution is expressed as a positive semi-definite bilinear form in the Youla values, with coefficients given by population-weighted Plücker coordinates of the Youla two-planes. This gives an explicit way to identify what is lost when the aligned geometry is replaced by a general orientation.

The second objective is to turn the hierarchical identity into a genuinely information-theoretic statement. For an aligned two-level block, increasing the Youla value does not simply increase the quadratic purity contribution. It sharpens the two eigenvalues of the block in the sense of majorization. Consequently, for fixed intrinsic populations and fixed pairing, increasing any aligned intrinsic coherence monotonically decreases every Rényi entropy, including the von Neumann entropy in the limit. This result interprets intrinsic coherence as a mechanism for converting diagonal population uncertainty into spectral concentration. A complementary optimization theorem shows that, for fixed ordered populations, the maximum aligned cohesion is obtained by pairing adjacent populations and saturating the corresponding Youla values. Thus, the extremal aligned problem reduces to a maximum-weight matching problem with an explicit solution.

The third objective is structural. Orthonormal eigenstate sets obey a sum rule for their antisymmetric blocks. In dimension three, this recovers the familiar coplanarity-type constraint for the corresponding antisymmetric descriptors; in higher dimensions, it becomes a constraint on tuples of real antisymmetric matrices and their joint Youla structure. This provides a dimension-independent algebraic way to describe eigenstate geometry without relying on a special vector representation available only in three dimensions.

Finally, this article revisits the dimensional transition in the cohesion bound of the discriminating component. The discriminating decomposition splits ρ into a pure component, a discriminating component, and a maximally mixed component; the discriminating component is the term that carries the structural information distinguishing states beyond their degree of purity [13,15]. Nonregularity is used here strictly in the discriminating sense. A state is nonregular when its discriminating component is not real, i.e., when it retains a nonzero purely imaginary antisymmetric part and the imaginary content of the state is not exhausted by the pure component. The known transition from $N_{3}=1/\left(2\sqrt{2}\right)$ to $N_{n\geq 4}=1/2$ [13] is not reproved here; rather, it is reinterpreted algebraically as a transition from one to two simultaneously saturating metaspin pairs. This interpretation explains why a scalar cohesion descriptor hides the structural change while the hierarchical identity resolves it.

The logical dependencies among the main constructions are summarized by the schematic chain

(2) $$\begin{array}{cc} \rho & ⟶\rho_{O}=A+iN,\\ A & ⟶\{M_{k}\},\\ \left(A,N\right) & ⟶M,\\ M & ⟶\left(\left\{s_{k}\right\},Q_{M}\right)⟶\left(\left\{C_{k}\right\},G,\Lambda_{\mathrm{IRB}}\right). \end{array}$$

followed, in the aligned class, by the block spectra (49), the majorization theorem, and the entropic cohesion (62). Thus, the later quantities are not independent assumptions. They are successive refinements of the same intrinsic decomposition. Along this chain, *A* controls population asymmetry, *N* controls intrinsic imaginary coherence, the metaspin tensor separates amplitude from population scale, and the Plücker matrix *G* records the remaining orientation of the Youla planes relative to the IRB.

The article is organized as follows. Section 2 recalls the IRB, the indices of population asymmetry, the metaspin tensor, and the scalar complementarity identity. Section 3 introduces the aligned class, proves the hierarchical identity, gives the bilinear-form extension to arbitrary orientation, and establishes the maximum aligned-cohesion theorem. Section 4 derives the eigenstate sum rule and its structural consequences. Section 5 develops the Rényi-α reformulation, proves spectral sharpening by majorization, and introduces entropic cohesion as the entropy reduction relative to the intrinsic population distribution. Section 6 reinterprets the dimensional transition in the discriminating-component cohesion bound. Section 7 gives worked examples, including an explicit two-qubit depolarized Bell state, representative aligned families in dimension four, and the five-dimensional embedding of the doubly coherent extremal configuration. Section 8 discusses the role of the framework in quantum information, state-space geometry, and potential interfaces with resource theories of imaginarity relative to a fixed real structure. Appendix A gives the explicit four-dimensional bilinear form using the decomposition $SO\left(4\right)≅\left(SU\left(2\right)\times SU\left(2\right)\right)/Z_{2}$.

## 2. The Intrinsic Reference Basis and Its Hierarchical Descriptors

This section fixes notation and recalls the constructions on which the rest of the article builds. The presentation follows Refs. [13,16], to which the reader is referred for proofs and detailed discussion.

### 2.1. The Intrinsic Reference Basis

Throughout this article, n≥2, and all logarithms are natural logarithms unless explicitly stated otherwise. Let ρ be an *n*-dimensional density matrix, that is, a Hermitian, positive semi-definite, trace-normalized n×n matrix. Decomposing ρ into its real symmetric and antisymmetric parts gives ρ=S+iT, with $S=(\rho +\bar{\rho })/2$ symmetric and $T=\left(\rho -\bar{\rho }\right)/\left(2i\right)$ antisymmetric. Because *S* is real symmetric, there exists a real orthogonal matrix $Q_{O}\in O\left(n\right)$ that diagonalizes it; an orientation convention may be imposed separately when required. Applying the same congruence to ρ yields the *intrinsic representation*

(3) $$\rho_{O}=Q_{O}^{T}\rho \;Q_{O}=A+iN,\;A=\mathrm{diag}\left(a_{1},\ldots ,a_{n}\right),\;a_{1}\geq a_{2}\geq \cdots \geq a_{n}\geq 0,\;N^{T}=-N,$$

in which *A* is diagonal and *N* is real antisymmetric. The basis defined by the columns of $Q_{O}$ is the *intrinsic reference basis* (IRB) of ρ. The convention adopted here, $\rho_{O}=A+iN$ without a prefactor on the antisymmetric block, is the one used throughout the recent literature on intrinsic representations of higher-dimensional density matrices [13,16]; the alternative $\rho_{O}=A+\left(i/2\right)\tilde{N}$ employed in earlier work [15] differs by a factor of two in the antisymmetric block, with $\tilde{N}=2N$. For the fixed real structure, the intrinsic populations $a_{i}$ and the antisymmetric coherence block *N* are covariant data under real-orthogonal conjugation of ρ; after applying the correspondingly transformed IRB, the intrinsic representation is unchanged up to ordering, sign choices, and residual rotations inside degenerate subspaces. They are not invariants under arbitrary complex unitary similarities, which need not preserve the real–imaginary split. When the real part *S* has degenerate eigenvalues, the IRB is not unique inside the degenerate subspaces. All statements involving a particular pairing of axes, a Youla orientation, or an alignment defect are therefore to be read either for the nondegenerate case, or after fixing a representative of the residual orthogonal freedom in each degenerate eigenspace. Quantities such as ${∥N∥}_{F}$ and the bilinear form written in invariant Plücker form remain well defined, whereas labels of individual pairs may change under the residual freedom.

The nonnegativity of the principal minors of ρ implies that the intrinsic coherences are constrained by the intrinsic populations through the inequalities $N_{ij}^{2}\leq a_{i}a_{j}$, with equality saturable only when the corresponding two-dimensional sector is internally pure. These pairwise inequalities are basic algebraic constraints on the intrinsic representation; they are necessary consequences of the positivity of ρ but do not exhaust it, since positive semi-definiteness imposes further joint constraints beyond the individual two-dimensional sectors.

### 2.2. Indices of Population Asymmetry

The intrinsic populations $a_{i}$ admit a hierarchical reorganization into the n−1 *indices of population asymmetry* [14,15],

(4) $$M_{k}=\sum_{j=1}^{k}a_{j}-k\;a_{k+1},\;k=1,\ldots ,n-1,$$

which satisfy the nested inequalities $0\leq M_{1}\leq M_{2}\leq \cdots \leq M_{n-1}\leq 1$. The value $M_{k}$ measures the cumulative excess of the first *k* populations over *k* times the (k+1)-th population; in particular, $M_{n-1}=1$ if and only if $a_{n}=0$, i.e., if and only if the intrinsic population matrix *A* is rank-deficient. A pure state obeys the stronger condition $a_{1}=1$ and $a_{2}=\cdots =a_{n}=0$. At the opposite endpoint, $M_{1}=M_{2}=\cdots =M_{n-1}=0$ corresponds to the maximally mixed state. The indices $M_{k}$ are determined by the populations and the ordering choice fixed by (3).

The squared norm of the population vector admits the explicit expression

(5) $$\sum_{i=1}^{n}a_{i}^{\;2}=\frac{1}{n}+\sum_{k=1}^{n-1}\frac{M_{k}^{\;2}}{k(k+1)},$$

which is a standard identity for the magnitudes of a probability simplex under the Bloch parametrization. The *degree of population asymmetry* is defined accordingly as

(6) $$P_{p}^{\;2}=\frac{n}{n-1}\sum_{k=1}^{n-1}\frac{M_{k}^{\;2}}{k(k+1)},$$

so that $0\leq P_{p}\leq 1$ and $P_{p}$ is monotonically increasing in each $M_{k}$.

### 2.3. The Metaspin Tensor and Indices of Correlation Asymmetry

The antisymmetric block *N* of the intrinsic representation admits a dimensionless rescaling that captures its geometric content in a way that is intrinsic to the IRB. For states with strictly positive populations, the *metaspin tensor* [16] (there denoted M) is the real antisymmetric matrix M with entries

(7) $$M_{ij}=\frac{N_{ij}}{\sqrt{a_{i}\;a_{j}}},\;i\neq j,$$

For states with zero intrinsic populations, positivity implies that every row and column of *N* associated with a zero population vanishes. The tensor is then defined by (7) on the support of *A* and extended by zeros to the inactive subspace; equivalently, it is obtained by using the Moore–Penrose pseudoinverse of $A^{1/2}$, as detailed in Ref. [16]. The inequalities $N_{ij}^{\;2}\leq a_{i}a_{j}$ translate into the bound $|M_{ij}|\leq 1$ at the level of individual entries. A stronger operator bound follows directly from positivity. On the support of *A*, one may write

(8) $$\rho_{O}=A^{1/2}\left(I+iM\right)A^{1/2},$$

so that $\rho_{O}\geq 0$ is equivalent, on that support, to I+iM≥0. Since the eigenvalues of iM are $\pm s_{k}$ (and one additional zero when *n* is odd), this gives $1-s_{k}\geq 0$ for every Youla value. Hence, all Youla values satisfy $0\leq s_{k}\leq 1$. The global Frobenius bound ${∥M∥}_{F}\leq \sqrt{2\lfloor n/2\rfloor }$ then follows from ${∥M∥}_{F}^{2}=2\sum_{k}s_{k}^{2}$.

A real antisymmetric matrix admits a Youla canonical form in which it is block-diagonalized by a real orthogonal congruence into two-dimensional rotation blocks [17]. Specifically, there exists $Q_{M}\in O\left(n\right)$ such that

(9) $$Q_{M}^{T}M\;Q_{M}=\overset{\left\lfloor n/2\right\rfloor }{\underset{k=1}{⨁}}\left(\begin{array}{cc} 0 & s_{k}\\ -s_{k} & 0 \end{array}\right)⊕0_{n-2\lfloor n/2\rfloor },$$

where the *Youla values* $s_{k}$ satisfy $1\geq s_{1}\geq s_{2}\geq \cdots \geq s_{\left\lfloor n/2\right\rfloor }\geq 0$.

The Youla values admit a hierarchical reorganization parallel to that of the intrinsic populations, the *indices of correlation asymmetry* [16],

(10) $$C_{k}=\sum_{j=1}^{k}s_{j}-k\;s_{k+1},\;k=1,\ldots ,\left\lfloor n/2\right\rfloor ,$$

with the convention $s_{\lfloor n/2\rfloor +1}\equiv 0$. The $C_{k}$ satisfy the nested inequalities $0\leq C_{1}\leq C_{2}\leq \cdots \leq C_{\left\lfloor n/2\right\rfloor }$ and provide a complete description of the Youla spectrum of the metaspin tensor under orthogonal congruence. They do not, by themselves, encode the relative orientation of the Youla two-planes with respect to the IRB axes. That additional information enters the general bilinear form of Section 3.6. A compact scalar summary of correlation content is provided by the mean-square Youla value [16],

(11) $$P_{M}^{\;2}=\frac{{∥M∥}_{F}^{\;2}}{2\lfloor n/2\rfloor }=\frac{1}{\left\lfloor n/2\right\rfloor }\sum_{k=1}^{\left\lfloor n/2\right\rfloor }s_{k}^{\;2},$$

a dimensionless invariant bounded by $0\leq P_{M}\leq 1$, in direct analogy with the degree of population asymmetry $P_{p}$ of (6). Because $P_{M}$ is a scalar, it does not record how the correlation content is distributed across Youla pairs. The indices $C_{k}$ supply this information.

### 2.4. Scalar Complementarity Formula

The Frobenius norm of ρ admits the decomposition $\mathrm{tr}\left(\rho^{2}\right)={∥A∥}_{F}^{\;2}+{∥N∥}_{F}^{\;2}$ in the intrinsic representation, which together with ${∥A∥}_{F}^{\;2}=\sum_{i}a_{i}^{\;2}$ and (5) expresses the *normalized degree of purity* of ρ,

(12) $$P_{nD}^{\;2}=\frac{n\;\mathrm{tr}(\rho^{2})-1}{n-1},$$

as a sum of two nonnegative contributions. Defining the *degree of correlation asymmetry* [16] by

(13) $$P_{c}^{\;2}=2\;{∥N∥}_{F}^{\;2}=4\sum_{i<j}N_{ij}^{\;2},$$

the decomposition takes the form

(14) $$P_{nD}^{\;2}=P_{p}^{\;2}+\frac{n}{2(n-1)}\;P_{c}^{\;2},$$

known as the scalar complementarity identity. The two contributions account for the two independent algebraic sources of purity, namely the asymmetry between populations and the asymmetry between conjugate pairs encoded in the antisymmetric block. The prefactor in (13) is fixed so that the numerical value of $P_{c}$ coincides with that of the degree of correlation asymmetry of Refs. [15,16] despite the different conventions for *N*.

The cohesion of the antisymmetric block, ${∥N∥}_{F}$, is itself bounded by a dimension-dependent constant when restricted to the discriminating component of ρ. The bound is [13]

(15) $$∥N\left(\rho_{d}\right){∥}_{F}\leq N_{n},\;N_{3}=\frac{1}{2\sqrt{2}},\;N_{n\geq 4}=\frac{1}{2},$$

where $\rho_{d}$ denotes the discriminating component of the discriminating decomposition [15]. In this terminology, nonregularity does not mean merely that N≠0 in the IRB. It means that the discriminating component itself is not real. Equivalently, the state has a nonzero purely imaginary contribution in $\rho_{d}$, so that the imaginary content of the state is not exhausted by the pure component of the decomposition. The discontinuity by a factor of $\sqrt{2}$ between n=3 and n=4 reflects the dimensional transition between single-pair and doubly coherent saturating configurations of the metaspin tensor of the discriminant, a structural feature that will reappear in the hierarchical refinement of the present article and will be revisited from the algebraic side in Section 6.

## 3. Hierarchical Complementarity Identity in the Aligned Class

The scalar complementarity identity (14) combines all of the imaginary cohesion of ρ into the single global descriptor $P_{c}^{\;2}$. This formulation is complete at the scalar level, but it loses resolution in dimensions four and higher, where the imaginary cohesion can be distributed across multiple intrinsic principal pairs. The present section refines the scalar identity by retaining the pair-by-pair structure of the antisymmetric block, under a structural restriction that defines a nontrivial subclass of states.

### 3.1. The Aligned Class

The Youla decomposition (9) of the metaspin tensor is generally achieved by an orthogonal congruence $Q_{M}$ that does not coincide with the orientation of the IRB itself. The angular content of $Q_{M}$ relative to the IRB axes provides additional structural information about ρ beyond the populations and the Youla values.

**Definition 1.**  *A density matrix ρ belongs to the* aligned class *when there exists a representative of its IRB, including the residual real-orthogonal freedom inside degenerate eigenspaces of A, in which the Youla decomposition of its metaspin tensor is realized by a signed permutation of the IRB axes. Equivalently, in at least one admissible IRB representative, there exists a permutation π of {1,…,n} such that the entries $M_{ij}$ vanish unless {i,j}={π(2k−1),π(2k)} for some k∈{1,…,⌊n/2⌋}.*

For a state in the aligned class, choose an admissible aligned IRB representative. In that representative, the metaspin tensor is supported on ⌊n/2⌋ disjoint pairs of intrinsic axes, the *aligned Youla pairs* $\left(i_{k},j_{k}\right)$ with $\left\{i_{k},j_{k}\right\}=\left\{\pi \left(2k-1\right),\pi \left(2k\right)\right\}$ and $i_{k}<j_{k}$, and the values $M_{i_{k}j_{k}}$ coincide up to signs with the Youla values $s_{k}$ of (9). The existence of such a representative is invariant under the residual freedom of the IRB and under signed permutations of its axes. Individual pair labels need not be unique when populations or Youla values are degenerate.

The aligned class contains a number of structurally significant configurations. The perfectly nonregular discriminating components of every dimension belong to it. The dimension-three maximizer has its single saturating Youla pair on the degenerate population pair $\left(a_{2},a_{3}\right)$ of the discriminant, while the dimension-four doubly coherent maximizer has two saturating Youla pairs on the two degenerate sectors $\left(a_{1},a_{2}\right)$ and $\left(a_{3},a_{4}\right)$. Here and below, nonregularity is understood in the discriminating sense stated above, not as the mere presence of an antisymmetric block in the total state. The aligned class also contains pure and mixed states whose antisymmetric blocks are supported on fixed disjoint IRB pairs. General Youla orientations are treated by the bilinear form of Section 3.6, while departures from matching-supported alignment are analyzed by the alignment defect of Section 3.9.

### 3.2. The Hierarchical Identity

Within the aligned class, the antisymmetric block of the intrinsic representation factorizes pair by pair, and the scalar identity (14) refines into a sum over the aligned Youla pairs weighted by the product of the corresponding intrinsic populations.

**Theorem 1.**  *Let ρ be a density matrix in the aligned class, represented in a selected aligned IRB representative, with aligned Youla pairs $\left(i_{k},j_{k}\right)$ and Youla values $s_{k}$ for k=1,…,⌊n/2⌋. The total normalized degree of purity admits the hierarchical decomposition*

(16) $$P_{nD}^{\;2}=\frac{n}{n-1}\left[\;\sum_{k=1}^{n-1}\frac{M_{k}^{\;2}}{k(k+1)}+2\sum_{k=1}^{\left\lfloor n/2\right\rfloor }a_{i_{k}}\;a_{j_{k}}\;s_{k}^{\;2}\right].$$

The decomposition (16) exhibits the dual hierarchical structure of the intrinsic representation. The first sum runs over n−1 population levels and reproduces $P_{p}^{\;2}$ as in (6); the second sum runs over ⌊n/2⌋ correlation levels, with each level contributing a term in which a Youla value $s_{k}$ is weighted by the product of the populations of the corresponding aligned pair. The two hierarchies have different lengths, and there are n−1−⌊n/2⌋ population levels that have no correlation counterpart; these levels contribute to the normalized purity solely through their population asymmetries.

**Proof of Theorem 1.**  The proof combines a Frobenius-norm decomposition of ρ in the IRB with the structural restriction imposed by alignment.

Since $\rho_{O}=A+iN$ is the IRB form of ρ and *A*, *N* are real with *A* symmetric and *N* antisymmetric, the cross terms tr(AN) and tr(NA) vanish, and the Frobenius norm of ρ admits the decomposition

(17) $$\mathrm{tr}\left(\rho^{2}\right)=\mathrm{tr}\left(\rho_{O}^{2}\right)={∥A∥}_{F}^{\;2}+{∥N∥}_{F}^{\;2}.$$

The population part satisfies, by the identity (5),

(18) $${n∥A∥}_{F}^{\;2}-1=n\sum_{i=1}^{n}a_{i}^{\;2}-1=\sum_{k=1}^{n-1}\frac{n\;M_{k}^{\;2}}{k(k+1)},$$

which divided by n−1 yields $P_{p}^{\;2}$ as in (6).

The correlation part is evaluated through the alignment hypothesis. For a state in the aligned class, the only nonzero entries of *N* (above the diagonal) are those at the aligned Youla pairs, $N_{i_{k}j_{k}}$ for k=1,…,⌊n/2⌋. By the definition of the metaspin tensor (7) and the alignment condition, the metaspin tensor entry on the *k*-th Youla pair coincides with the corresponding Youla value up to a sign, so

(19) $$N_{i_{k}j_{k}}^{\;2}=a_{i_{k}}\;a_{j_{k}}\;M_{i_{k}j_{k}}^{\;2}=a_{i_{k}}\;a_{j_{k}}\;s_{k}^{\;2}.$$

Since *N* is antisymmetric and supported on the aligned pairs, its Frobenius norm satisfies

(20) $${∥N∥}_{F}^{\;2}=2\sum_{i<j}N_{ij}^{\;2}=2\sum_{k=1}^{\left\lfloor n/2\right\rfloor }a_{i_{k}}\;a_{j_{k}}\;s_{k}^{\;2}.$$

Combining (17), (18), and (20), the normalized purity (12) reads

(21) $$P_{nD}^{\;2}=\frac{n\;\mathrm{tr}(\rho^{2})-1}{n-1}=\frac{{n∥A∥}_{F}^{\;2}-1}{n-1}+\frac{{n∥N∥}_{F}^{\;2}}{n-1},$$

and substituting the two sums gives the claimed identity (16). □

### 3.3. Recovery of the Scalar Complementarity Formula

The hierarchical identity (16) reduces to the scalar complementarity Formula (14) when the second sum is replaced by the global descriptor $P_{c}^{\;2}$ defined in (13). Indeed, in the aligned class, one has, from (20),

(22) $$P_{c}^{\;2}=2{∥N∥}_{F}^{\;2}=4\sum_{k=1}^{\left\lfloor n/2\right\rfloor }a_{i_{k}}\;a_{j_{k}}\;s_{k}^{\;2},$$

so that the second sum in (16) equals $P_{c}^{\;2}/2$, and the prefactor n/(n−1) multiplying it gives $n/\left(2\left(n-1\right)\right)\cdot P_{c}^{\;2}$ as in (14). The hierarchical identity is therefore an information-preserving refinement of the scalar one. The scalar formula is recovered by summing the correlation contributions across Youla pairs, while the hierarchical formula retains the distribution of contributions across pairs.

### 3.4. Maximum Aligned Cohesion for Fixed Populations

The aligned-class formula also yields a useful extremal result. The ordered intrinsic populations $a_{1}\geq a_{2}\geq \cdots \geq a_{n}\geq 0$ are fixed, and both the aligned pairing and the Youla values are allowed to vary, subject only to positivity. The largest aligned cohesion is obtained by pairing adjacent populations and saturating every active pair.

**Theorem 2.**  *For fixed ordered intrinsic populations $\left(a_{1},\ldots ,a_{n}\right)$, the maximum of ${∥N∥}_{F}^{2}$ over the aligned class is*

(23) $${∥N∥}_{F,\max}^{2}=2\sum_{k=1}^{\left\lfloor n/2\right\rfloor }a_{2k-1}a_{2k}.$$

*It is attained by the adjacent pairing (1,2),(3,4),… with $s_{k}=1$ for every paired sector satisfying $a_{2k-1}a_{2k}>0$; pairs with zero population product make no contribution and are inactive under the pseudoinverse convention. Equivalently,*

(24) $${∥N∥}_{F,\max}^{2}=2\max_{M}\sum_{(i,j)\in M}a_{i}a_{j},$$

*where the maximum is over all matchings M of ⌊n/2⌋ disjoint pairs of indices.*

**Proof.**  For an aligned state, positivity implies $0\leq s_{k}\leq 1$, and therefore,

(25) $${∥N∥}_{F}^{2}=2\sum_{(i,j)\in M}a_{i}a_{j}s_{ij}^{2}\leq 2\sum_{(i,j)\in M}a_{i}a_{j}.$$

For a fixed matching, equality is attained by $s_{ij}=1$ for every pair with $a_{i}a_{j}>0$; zero-weight pairs are inactive and do not affect the value. It remains to maximize the weight of the matching. Let $a_{i}\geq a_{j}\geq a_{k}\geq a_{l}$. The exchange inequality

(26) $$a_{i}a_{j}+a_{k}a_{l}-\left(a_{i}a_{k}+a_{j}a_{l}\right)=\left(a_{i}-a_{l}\right)\left(a_{j}-a_{k}\right)\geq 0$$

shows that pairing the two largest among four ordered entries and the two smallest among them is never worse than the crossing pairing (i,k),(j,l). Similarly,

(27) $$a_{i}a_{j}+a_{k}a_{l}-\left(a_{i}a_{l}+a_{j}a_{k}\right)=\left(a_{i}-a_{k}\right)\left(a_{j}-a_{l}\right)\geq 0$$

shows that it is never worse than the outer pairing (i,l),(j,k). Repeated exchanges transform any maximum-weight matching into the adjacent matching without decreasing its weight. If *n* is odd, the remaining unpaired entry must be the smallest population, since exchanging an unpaired larger entry with a paired smaller one cannot reduce the matching weight. This proves (23). □

The theorem gives a variational interpretation of the aligned sector. For a fixed intrinsic population profile, the largest possible imaginary cohesion is obtained by concentrating saturated metaspin correlations into adjacent population pairs. In this sense, the aligned class is not only algebraically tractable; it contains the extremal configurations of the fixed-population coherence problem restricted to aligned supports. Whether the same maximum is globally optimal over arbitrary Youla orientations is a separate optimization problem and is not assumed here.

### 3.5. Dimension-Three Reduction

In dimension three, ⌊n/2⌋=1, so the aligned class admits a single Youla pair $\left(i_{1},j_{1}\right)$ and a single Youla value $s_{1}$. The hierarchical identity reads

(28) $$P_{3D}^{\;2}=\frac{3}{2}\left[\frac{M_{1}^{\;2}}{2}+\frac{M_{2}^{\;2}}{6}+2\;a_{i_{1}}\;a_{j_{1}}\;s_{1}^{\;2}\right],$$

which agrees with the three-dimensional expressions of Refs. [13,15]. For the perfectly nonregular three-dimensional discriminating component, a=(1/2,1/4,1/4), $\left(M_{1},M_{2}\right)=\left(1/4,1/4\right)$, $\left(i_{1},j_{1}\right)=\left(2,3\right)$, $s_{1}=1$, the right-hand side evaluates to (3/2)[1/32+1/96+2·(1/16)]=1/4, in agreement with the standard value $P_{3D}^{\;2}\left(\rho_{d}^{\max,3}\right)=1/4$.

In dimensions four and higher, ⌊n/2⌋≥2 and the correlation sum (16) acquires multiple terms, each weighted by a distinct product of intrinsic populations. The qualitative difference between n=3 and n≥4 in the structure of the correlation sum is the algebraic source of the dimensional transition (15), as will be made explicit in Section 6.

### 3.6. Bilinear Form for Arbitrary Orientation

The hierarchical identity of Theorem 1 is formulated for states in the aligned class, where the orthogonal congruence $Q_{M}$ realizing the Youla form of the metaspin tensor reduces to a signed permutation of the IRB axes. Outside the aligned class, the same algebraic argument that yields (16) produces a decomposition of the correlation contribution as a bilinear form in the Youla values, with coefficients that record the angular content of $Q_{M}$ relative to the IRB.

The columns ${\left\{q_{m}^{\pm }\right\}}_{m=1}^{\left\lfloor n/2\right\rfloor }$ of $Q_{M}$, grouped into the pairs $\left(q_{m}^{-},q_{m}^{+}\right)=\left(Q_{M}\;e_{2m-1},Q_{M}\;e_{2m}\right)$ that span the *m*-th Youla two-plane define the *Plücker coordinates*

(29) $$\pi_{ij}^{\left(m\right)}=Q_{M,i,2m-1}\;Q_{M,j,2m}-Q_{M,i,2m}\;Q_{M,j,2m-1},\;1\leq i<j\leq n,$$

which are the 2×2 minors of the rectangular matrix formed by the (2m−1)-th and (2m)-th columns of $Q_{M}$. The Plücker coordinates of a single Youla pair satisfy the normalization $\sum_{i<j}{\left(\pi_{ij}^{\left(m\right)}\right)}^{\;2}=1$ and identify the *m*-th Youla two-plane as a point in the Grassmannian Gr(2,n) [18].

**Theorem 3.**  *For any density matrix ρ with intrinsic populations $\left\{a_{i}\right\}$ and Youla values $\left\{s_{m}\right\}$, the cohesion squared of the antisymmetric block of the intrinsic representation admits the decomposition*

(30) $${∥N∥}_{F}^{\;2}=\sum_{m,m^{\prime }=1}^{\left\lfloor n/2\right\rfloor }G_{mm^{\prime }}\left(a,Q_{M}\right)\;s_{m}\;s_{m^{\prime }},$$

*where G is the symmetric positive semi-definite matrix with entries*

(31) $$G_{mm^{\prime }}=2\sum_{i<j}a_{i}\;a_{j}\;\pi_{ij}^{\left(m\right)}\;\pi_{ij}^{\left(m^{\prime }\right)}.$$

*In the aligned class, G is diagonal with $G_{mm}=2\;a_{i_{m}}a_{j_{m}}$, and the bilinear form* (30) *reduces to the correlation sum of Theorem 1.*

**Proof.**  The Youla decomposition $Q_{M}^{T}M\;Q_{M}={⨁}_{m}B\left(s_{m}\right)$ of (9) translates into the explicit form

(32) $$M_{ij}=\sum_{m=1}^{\left\lfloor n/2\right\rfloor }s_{m}\;\pi_{ij}^{\left(m\right)}$$

for the entries of the metaspin tensor in the IRB. Recalling that $N_{ij}=\sqrt{a_{i}a_{j}}\;M_{ij}$ and that ${∥N∥}_{F}^{\;2}=2\sum_{i<j}N_{ij}^{\;2}$ by antisymmetry, substitution yields

(33) $${∥N∥}_{F}^{\;2}=2\sum_{i<j}a_{i}\;a_{j}{\left(\sum_{m}s_{m}\;\pi_{ij}^{\left(m\right)}\right)}^{\;2}=\sum_{m,m^{\prime }}\left[2\sum_{i<j}a_{i}\;a_{j}\;\pi_{ij}^{\left(m\right)}\pi_{ij}^{\left(m^{\prime }\right)}\right]s_{m}\;s_{m^{\prime }},$$

which is (30) with *G* as defined. Symmetry of *G* in $\left(m,m^{\prime }\right)$ is immediate from (31). Positive semi-definiteness follows from the observation that for any vector $v\in R^{\left\lfloor n/2\right\rfloor }$,

(34) $$v^{T}G\;v=2\sum_{i<j}a_{i}a_{j}{\left(\sum_{m}v_{m}\pi_{ij}^{\left(m\right)}\right)}^{\;2}\geq 0.$$

In the aligned class, the (2m−1)-th and (2m)-th columns of $Q_{M}$ are the standard basis vectors $e_{i_{m}}$ and $e_{j_{m}}$, so $\pi_{ij}^{\left(m\right)}=\pm 1$ exactly when $\left\{i,j\right\}=\left\{i_{m},j_{m}\right\}$ and vanishes otherwise. Different pairs $m\neq m^{\prime }$ have disjoint supports, giving $G_{mm^{\prime }}=0$ for $m\neq m^{\prime }$ and $G_{mm}=2a_{i_{m}}a_{j_{m}}$. □

If the Youla spectrum contains degeneracies, the Youla frame $Q_{M}$ is not unique within the corresponding degenerate sector. The antisymmetric block *N* and the scalar value $s^{T}Gs={∥N∥}_{F}^{\;2}$ remain unambiguous, whereas individual plane labels and entries of *G* refer to a selected Youla representative. In the aligned class, the pair-resolved formulas use an admissible representative supported on the chosen IRB pairs; at degenerate endpoints, this representative is selected consistently by the structural family under consideration.

Theorem 3 replaces the aligned-class restriction of Theorem 1 by an explicit characterization of the correlation contribution valid for arbitrary ρ. The cost of leaving the aligned class is the appearance of off-diagonal entries $G_{mm^{\prime }}$ with $m\neq m^{\prime }$, which encode the angular content of the Youla congruence and quantify the geometric overlap between distinct Youla two-planes weighted by the population distribution. The aligned class is contained in the diagonal locus where these overlaps vanish. The converse does not hold in general, since nonaligned Youla planes can also be orthogonal with respect to the population-weighted metric, as made explicit in Appendix A.

The hierarchical identity (16) generalizes accordingly to

(35) $$P_{nD}^{\;2}=\frac{n}{n-1}\left[\;\sum_{k=1}^{n-1}\frac{M_{k}^{\;2}}{k(k+1)}+\sum_{m,m^{\prime }=1}^{\left\lfloor n/2\right\rfloor }G_{mm^{\prime }}\;s_{m}\;s_{m^{\prime }}\right],$$

valid for any density matrix in dimension *n* and reducing to Theorem 1 when the cross terms in *G* vanish.

### 3.7. Reformulation in Terms of Correlation Asymmetry Indices

The bilinear form of Theorem 3 is expressed in terms of the Youla values $s_{m}$, which form the spectrum of the metaspin tensor but are not themselves indexed in a hierarchical manner. A reformulation in terms of the indices of correlation asymmetry $C_{k}$ defined in (10) is obtained through the linear change in variables that relates the two sets of invariants.

Define the invertible staircase matrix $L\in R^{\left\lfloor n/2\rfloor \times \lfloor n/2\right\rfloor }$ by

(36) $$L_{kl}=\left\{\begin{array}{cc} 1, & l\leq k,\\ -k, & l=k+1,\\ 0, & l>k+1, \end{array}\right.$$

so that $C_{k}=\sum_{l}L_{kl}\;s_{l}$, with the convention $s_{\lfloor n/2\rfloor +1}=0$. The matrix *L* is invertible, with inverse $L^{-1}$ admitting an explicit form related to the inverse of the staircase pattern that defines the indices of normalized purity. Substituting $s=L^{-1}C$ into the bilinear form (30) yields

(37) $${∥N∥}_{F}^{\;2}=C^{T}HC,\;H={\left(L^{-1}\right)}^{T}\;G\;L^{-1},$$

where *H* is symmetric positive semi-definite and depends on the populations and on $Q_{M}$ through the same Plücker structure as *G*.

The reformulation (37) arranges the correlation contribution as a quadratic form in the hierarchical indices $C_{k}$, paralleling the role of the indices of population asymmetry in the expression (6) for $P_{p}^{\;2}$. The two sets of indices, $M_{k}$ and $C_{k}$, both nested by inequalities and both running over their respective levels, organize the population and correlation sectors of the intrinsic representation in formally analogous ways, and the hierarchical complementarity identity acquires the symmetric form

(38) $$P_{nD}^{\;2}=\frac{n}{n-1}\left[\;\sum_{k=1}^{n-1}\frac{M_{k}^{\;2}}{k(k+1)}+\sum_{k,l=1}^{\left\lfloor n/2\right\rfloor }H_{kl}\;C_{k}\;C_{l}\right].$$

The diagonal limit $H_{kl}=h_{k}\;\delta_{kl}$ corresponds to a class of states intermediate between the aligned class and the general case, in which the indices of correlation asymmetry contribute independently to the cohesion. A characterization of this intermediate class through structural conditions on $Q_{M}$ is an open question of immediate algebraic interest.

### 3.8. Rank Structure of the Bilinear Form

The matrix *G* of Theorem 3 admits a geometric interpretation through the bilinear pairing on the space of 2-forms over $R^{n}$. The Plücker coordinates $\left\{\pi^{\left(m\right)}\right\}$ define ⌊n/2⌋ unit vectors in $\Lambda^{2}\left(R^{n}\right)$, and the entries $G_{mm^{\prime }}$ are their pairwise inner products under the population-weighted form ${\langle \alpha ,\beta \rangle }_{a}=2\sum_{i<j}a_{i}a_{j}\alpha_{ij}\beta_{ij}$. Two structural consequences follow.

First, the matrix *G* is defined for the full selected Youla frame and is independent of whether a particular Youla value happens to vanish. To describe the coherence content actually present in the state, let $I_{\mathrm{act}}=\left\{m:s_{m}>0\right\}$ and let $G_{\mathrm{act}}={\left(G_{mm^{\prime }}\right)}_{m,m^{\prime }\in I_{\mathrm{act}}}$ be the active principal submatrix. When all intrinsic populations are strictly positive, the population-weighted form is positive definite on $\Lambda^{2}\left(R^{n}\right)$, and the rank of $G_{\mathrm{act}}$ equals the dimension of the span of the active Plücker vectors $\left\{\pi^{\left(m\right)}:m\in I_{\mathrm{act}}\right\}$. If some intrinsic populations vanish, the population-weighted form is only semi-definite; in that case, the rank of $G_{\mathrm{act}}$ equals the dimension of the images of the active vectors after quotienting by the null subspace generated by pairs with $a_{i}a_{j}=0$. Thus, *G* describes the selected ambient Youla frame, whereas $G_{\mathrm{act}}$ describes the two-plane content carried by nonzero coherence amplitudes.

Second, the off-diagonal of *G* admits a representation-intrinsic interpretation as the population-weighted overlap of distinct Youla two-planes. States in the aligned class have vanishing off-diagonal overlaps because their Plücker vectors are supported on disjoint pairs of intrinsic axes. This is a sufficient but not necessary condition for *G* to be diagonal, since nonaligned Youla planes may be mutually orthogonal under the population-weighted form. The magnitude of the off-diagonal entries of *G* therefore measures failure of weighted diagonality, whereas the stricter failure of support alignment is quantified below directly from the entries of *N*. A quantitative characterization of this interpolation, in the form of a scalar invariant of (ρ,IRB) that measures the departure from alignment, is developed in the following subsection.

### 3.9. Alignment Defect Modulo Residual IRB Freedom

If the intrinsic populations are nondegenerate, the ordered IRB is fixed up to independent sign reversals of its axes, and support alignment can be tested directly from the entries of *N*. If *A* has degenerate eigenspaces, however, the IRB has a residual orthogonal freedom that changes the entries of *N* while preserving the state-adapted population frame. A representation-intrinsic alignment descriptor must therefore optimize over this residual group.

Let

(39) $$G_{A}=\left\{R\in O\left(n\right):R^{T}AR=A\right\}$$

be the residual orthogonal group of the ordered intrinsic population matrix *A*. For a selected IRB representative with antisymmetric block *N*, first, define the fixed-frame unmatched cohesion

(40) $$\lambda_{\mathrm{fr}}\left(N\right)=\frac{1}{2}{∥N∥}_{F}^{\;2}-\max_{\sigma \in M_{n}}\sum_{(i,j)\in \sigma }N_{ij}^{\;2},$$

where $M_{n}$ denotes the set of maximum-cardinality matchings on the intrinsic axes. The intrinsic defect is the residual-orbit minimum.

**Definition 2.**  *The* residual-invariant alignment defect *of ρ is*

(41) $$\Lambda_{\mathrm{IRB}}\left(\rho \right)=\min_{R\in G_{A}}\lambda_{\mathrm{fr}}\left(R^{T}NR\right).$$

The minimum exists because $G_{A}$ is compact and $\lambda_{\mathrm{fr}}$ is continuous. In the nondegenerate case, $G_{A}$ consists only of independent axis sign changes after ordering has been fixed; these do not change squared entries, and therefore, $\Lambda_{\mathrm{IRB}}\left(\rho \right)=\lambda_{\mathrm{fr}}\left(N\right)$. In dimension four, the fixed-frame function appearing inside the minimization is

(42) $$\lambda_{\mathrm{fr}}\left(N\right)=\frac{1}{2}{∥N∥}_{F}^{\;2}-\max\;\left(N_{12}^{\;2}+N_{34}^{\;2},\;N_{13}^{\;2}+N_{24}^{\;2},\;N_{14}^{\;2}+N_{23}^{\;2}\right).$$

**Proposition 1.**  *The defect $\Lambda_{\mathrm{IRB}}\left(\rho \right)$ is nonnegative, is independent of the selected representative of the IRB, and vanishes if and only if ρ belongs to the aligned class of Definition 1.*

**Proof.**  For every antisymmetric matrix $N^{\prime }$ and every matching σ, one has $\frac{1}{2}{∥N^{\prime }∥}_{F}^{\;2}=\sum_{i<j}{\left(N_{ij}^{\prime }\right)}^{2}\geq \sum_{(i,j)\in \sigma }{\left(N_{ij}^{\prime }\right)}^{2}$; hence, $\lambda_{\mathrm{fr}}\left(N^{\prime }\right)\geq 0$ and, therefore, $\Lambda_{\mathrm{IRB}}\left(\rho \right)\geq 0$. Replacing the selected IRB representative by $R_{0}^{T}NR_{0}$ with $R_{0}\in G_{A}$ leaves the minimum unchanged because $R\mapsto R_{0}R$ permutes the compact optimization domain. Finally, the minimum is zero exactly when some $R\in G_{A}$ makes $R^{T}NR$ supported on a maximum matching of intrinsic axes. This is precisely the existence condition in Definition 1. □

The defect $\Lambda_{\mathrm{IRB}}\left(\rho \right)$ is the smallest unmatched cohesion squared among all representatives compatible with the intrinsic populations. It therefore quantifies the obstruction to rendering the antisymmetric support matching-aligned without changing the ordered population matrix. For a fixed representative, the maximizing matching in (40) identifies the matching-supported antisymmetric block capturing the largest squared cohesion weight, equivalently the closest matching-supported block to that representative in Frobenius norm. No claim is made that replacing *N* by such a truncation preserves positivity of the full density matrix.

The scaling of the intrinsic defect under dephasing is exact when dephasing is performed in a fixed representative of the IRB. This qualification is essential because the frame is state-adapted, and the operation below is not claimed to define a single state-independent resource-theoretic channel on the full state space.

**Proposition 2.**  *Let $Q_{O}$ be a selected IRB representative for ρ, so that $Q_{O}^{T}\rho Q_{O}=A+iN$, and define*

(43) $$E_{p}^{\left(Q_{O}\right)}\left(X\right)=\left(1-p\right)X+p\;Q_{O}\;\mathrm{diag}\left(Q_{O}^{T}XQ_{O}\right)\;Q_{O}^{T},\;p\in \left[0,1\right].$$

*Then,*

(44) $$\Lambda_{\mathrm{IRB}}\;\left(E_{p}^{\left(Q_{O}\right)}\left(\rho \right)\right)={\left(1-p\right)}^{\;2}\Lambda_{\mathrm{IRB}}\left(\rho \right).$$

**Proof.**  In the selected IRB, $Q_{O}^{T}E_{p}^{\left(Q_{O}\right)}\left(\rho \right)Q_{O}=A+i\left(1-p\right)N$. The residual group $G_{A}$ is unchanged. For every $R\in G_{A}$, homogeneity of (40) gives $\lambda_{\mathrm{fr}}\left(\left(1-p\right)R^{T}NR\right)={\left(1-p\right)}^{2}\lambda_{\mathrm{fr}}\left(R^{T}NR\right)$. Taking the minimum over $G_{A}$ yields (44). □

Proposition 2 gives a precise variational monotonicity statement for the residual-invariant defect under a specified state-adapted dephasing family. Identifying a state-independent class of completely positive trace-preserving maps under which $\Lambda_{\mathrm{IRB}}$ is monotonic remains an open problem.

## 4. Eigenstate Geometry and Structural Constraints

The hierarchical identity of Section 3 is formulated for a single density matrix and its intrinsic representation. A complementary line of structural analysis concerns the geometry of orthonormal sets of pure states, which arises in the spectral decomposition of an arbitrary density matrix and in the characterization of measurement bases for quantum protocols. In dimension three, the geometry of such sets has been characterized in detail in Ref. [19], where it was shown that the antisymmetric-vector representatives of three orthonormal pure states satisfy a coplanarity constraint and that two of the associated azimuthal parameters coincide once the first state is taken as reference. The present section establishes the general structural constraint that underlies these dimension-three results and that admits an immediate generalization to arbitrary dimension through the metaspin tensor framework.

### 4.1. The Eigenstate Sum Rule

Let ρ be an *n*-dimensional density matrix, and let $\{|u_{k}\rangle {\}}_{k=1}^{n}$ be an orthonormal set of pure states. The completeness relation $\sum_{k}|u_{k}\rangle \langle u_{k}|=I_{n}$, valid for any orthonormal basis of $C^{n}$, decomposes into real symmetric and antisymmetric parts:

(45) ∑k=1nRe|uk〉〈uk|=In,∑k=1nIm|uk〉〈uk|=0.

The first identity reflects the trace structure of orthonormal projectors. The second identity, the antisymmetric component of completeness, is the structural constraint that the present article identifies as the dimension-independent generalization of the spin-vector coplanarity of Ref. [19].

**Theorem 4.**  *For any orthonormal set $\{|u_{k}\rangle {\}}_{k=1}^{n}$ of pure states in dimension n, the antisymmetric parts of the corresponding rank-one projectors, expressed in any common basis, sum to zero.*

The theorem is an immediate consequence of the orthonormality relation $\sum_{k}|u_{k}\rangle \langle u_{k}|=I_{n}$ and the fact that $I_{n}$ is real. Its structural content lies in the implication that the antisymmetric blocks of the eigenstates, viewed as elements of the n2-dimensional space of real antisymmetric matrices, are linearly dependent. The *n* matrices N(k)=Im(|uk〉〈uk|) span at most an (n−1)-dimensional subspace, and any one of them is determined by the remaining n−1.

### 4.2. Recovery of the Three-Dimensional Coplanarity

In dimension three, every real antisymmetric matrix is dual to a vector in $R^{3}$. One may write $N_{ij}^{\left(k\right)}=-\frac{1}{2}\varepsilon_{ijl}n_{l}^{\left(k\right)}$, which defines the spin vector $n^{\left(k\right)}$ of each eigenstate. The sum rule (45) translates into the vector identity

(46) $$\sum_{k=1}^{3}n^{\left(k\right)}=0,$$

which expresses the coplanarity of the three vectors associated with any orthonormal set of pure states [19]. Three vectors summing to zero in $R^{3}$ lie in the plane spanned by any two linearly independent members of the set; equivalently, the normal to the plane is proportional to $n^{\left(1\right)}\times n^{\left(2\right)}$ when this cross product is nonzero. The coplanarity constraint of Ref. [19] is therefore an immediate dimensional reduction of Theorem 4, and the related azimuth-sharing property $\phi_{3}=\phi_{2}$ of the second and third eigenstates relative to the first is a parametric consequence of the same constraint in the coordinates used in that reference.

### 4.3. Structural Consequences in Higher Dimensions

For n≥4, the antisymmetric matrices $N^{\left(k\right)}$ no longer admit a canonical axial-vector representation in the underlying real space. They are bivectors in $\Lambda^{2}\left(R^{n}\right)$, of dimension n2. The special Hodge identification $\Lambda^{2}\left(R^{3}\right)≃R^{3}$ that makes a single spin-vector picture possible is no longer available. The metaspin tensor of each eigenstate, defined as in (7) with respect to the eigenstate’s own intrinsic populations, organizes the geometric content in a way that is amenable to a hierarchical analysis paralleling that of Section 3.

Two structural consequences of Theorem 4 in dimensions four and higher are worth recording.

First, the linear constraint $\sum_{k}N^{\left(k\right)}=0$ is a single matrix equation amounting to n2 scalar linear relations among the antisymmetric blocks. A complete dimension count of the admissible tuple space would also have to impose the nonlinear orthonormality constraints on the underlying pure states; no such count is needed for the sum rule, and none is asserted here.

Second, when ρ belongs to the aligned class of Section 3, the antisymmetric block *N* of ρ has Youla support on the aligned pairs $\left(i_{k},j_{k}\right)$, and the sum rule (45), weighted by the eigenvalues $\lambda_{k}$ of ρ, gives

(47) $$\sum_{k=1}^{n}\lambda_{k}\;N^{\left(k\right)}=N,\;\sum_{k=1}^{n}N^{\left(k\right)}=0.$$

The two relations, taken together, imply that the eigenstate antisymmetric blocks $N^{\left(k\right)}$ are organized into a structure compatible with the aligned-pair support of *N*. The Youla support of each $N^{\left(k\right)}$ need not coincide with that of *N*, but the linear combinations (47) require a coordinated arrangement of the eigenstate antisymmetric blocks. A detailed characterization of this arrangement, including the relation to the indices of correlation asymmetry of the individual eigenstates, lies beyond the scope of the present article and is left for future work.

### 4.4. Connection to the Hierarchical Identity

The hierarchical complementarity identity of Theorem 1 and the eigenstate sum rule of Theorem 4 are complementary characterizations of the structural content of the intrinsic representation. The former decomposes the normalized purity of a single density matrix into level-by-level contributions controlled by the population and correlation hierarchies. The latter constrains the antisymmetric blocks of orthonormal sets of pure states through a linear identity that does not depend on the spectrum or on the alignment status of any specific ρ. The two results together provide a representation-intrinsic infrastructure for the analysis of *n*-dimensional density matrices that is invariant under real orthogonal conjugation and that recovers the dimension-three results of Refs. [15,19] as specific limits.

## 5. Rényi-α Reformulation

The hierarchical identity (16) is quadratic in the population and correlation invariants. Quadratic identities of this form are natural counterparts of the squared Frobenius norm of ρ and connect to the linear entropy through the relation $S_{\mathrm{lin}}\left(\rho \right)=1-\mathrm{tr}\left(\rho^{2}\right)$. A continuous family of related identities, parametrized by a Rényi index α, interpolates between the quadratic regime and the von Neumann entropy and clarifies the information-theoretic content of the intrinsic representation.

### 5.1. Spectrum of an Aligned-Class State

Within the aligned class, the intrinsic representation $\rho_{O}$ decomposes into 2×2 blocks supported on the aligned Youla pairs together with 1×1 blocks on the unpaired axes. Each 2×2 block has the form

(48) $$\rho_{O}^{\left(k\right)}=\left(\begin{array}{cc} a_{i_{k}} & i\sqrt{a_{i_{k}}a_{j_{k}}}\;s_{k}\\ -i\sqrt{a_{i_{k}}a_{j_{k}}}\;s_{k} & a_{j_{k}} \end{array}\right),$$

with eigenvalues

(49) $$\lambda_{\pm }^{\left(k\right)}=\frac{a_{i_{k}}+a_{j_{k}}}{2}\pm \frac{1}{2}\sqrt{{\left(a_{i_{k}}-a_{j_{k}}\right)}^{\;2}+4a_{i_{k}}a_{j_{k}}\;s_{k}^{\;2}}.$$

The unpaired axes contribute eigenvalues equal to the corresponding intrinsic populations $a_{l}$. The spectrum of ρ is therefore given explicitly by the union of these pair eigenvalues and unpaired populations, depending only on $\left(\left\{a_{i}\right\},\left\{s_{k}\right\}\right)$ and on the pairing assignment $k\mapsto (i_{k},j_{k})$.

Two limiting cases of (49) are relevant. When $s_{k}=0$, the block is real and diagonal, with eigenvalues $a_{i_{k}}$ and $a_{j_{k}}$ unchanged from the populations; this is the IRB-real, or coherence-free, limit of the pair. When $s_{k}=1$, the saturation $N_{i_{k}j_{k}}^{\;2}=a_{i_{k}}a_{j_{k}}$ implies $\lambda_{+}^{\left(k\right)}=a_{i_{k}}+a_{j_{k}}$ and $\lambda_{-}^{\left(k\right)}=0$, corresponding to a rank-one pair contribution to the state.

### 5.2. Spectral Sharpening by Intrinsic Coherence

The block spectrum (49) implies a stronger statement than the quadratic identity. For fixed populations and fixed aligned pairing, increasing the Youla values makes the spectrum more ordered in the sense of majorization. This gives a rigorous entropic meaning to intrinsic coherence.

For a pair $\left(i_{k},j_{k}\right)$ with $u_{k}=a_{i_{k}}+a_{j_{k}}>0$, define

(50) $$\delta_{k}=\frac{|a_{i_{k}}-a_{j_{k}}|}{u_{k}},\;r_{k}\left(s_{k}\right)=\sqrt{\delta_{k}^{2}+\left(1-\delta_{k}^{2}\right)s_{k}^{2}}.$$

Then, the two eigenvalues of the block are

(51) $$\lambda_{\pm }^{\left(k\right)}=\frac{u_{k}}{2}\left(1\pm r_{k}\left(s_{k}\right)\right).$$

Since $r_{k}\left(s_{k}\right)$ is nondecreasing in $s_{k}$, the larger eigenvalue increases and the smaller one decreases while their sum remains fixed.

This use of majorization should be distinguished from state-conversion criteria in quantum resource theories. In Nielsen’s theorem, for example, majorization orders pure bipartite states under deterministic local operations and classical communication [20]. Here, majorization has a different role. It orders spectra along a fixed intrinsic-population fibre. The populations and pairing are held fixed, and only the intrinsic antisymmetric amplitudes are increased. The result therefore gives a spectral and entropic interpretation of Youla coherence, not a general convertibility criterion under a prescribed class of free operations.

**Theorem 5.**  *Let ρ(s) and $\rho (s^{\prime })$ be aligned-class states with the same intrinsic populations and the same aligned pairing but with Youla values satisfying $s_{k}^{\prime }\geq s_{k}$ for every active pair. Then,*

(52) $$\lambda \left(\rho \left(s^{\prime }\right)\right)≻\lambda \left(\rho \left(s\right)\right),$$

*where ≻ denotes majorization of spectra. Consequently, for every Rényi order α>0, including the von Neumann limit α→1,*

(53) $$S_{\alpha }\left(\rho \left(s^{\prime }\right)\right)\leq S_{\alpha }\left(\rho \left(s\right)\right).$$

*The inequality is strict for strictly Schur-concave entropies whenever at least one pair with $a_{i_{k}}a_{j_{k}}>0$ has $s_{k}^{\prime }>s_{k}$.*

**Proof.**  For a single two-dimensional block, the two spectra have the same sum $u_{k}$. If $s_{k}^{\prime }\geq s_{k}$, then $r_{k}\left(s_{k}^{\prime }\right)\geq r_{k}\left(s_{k}\right)$, so the ordered pair of eigenvalues at $s_{k}^{\prime }$ majorizes the ordered pair at $s_{k}$. Direct sums preserve majorization [21], and therefore, if each block spectrum majorizes the corresponding block spectrum and the unpaired one-dimensional entries are unchanged, then the union of all block spectra of $\rho (s^{\prime })$ majorizes the union of all block spectra of ρ(s). This proves (52). Rényi entropies are Schur-concave for α>0 [21,22]; hence, (53) follows. □

For the von Neumann entropy, the entropy loss relative to the diagonal population distribution has an explicit binary form. With h(x)=−xlogx−(1−x)log(1−x), the contribution of the *k*-th pair is

(54) $$\Delta S^{\left(k\right)}=u_{k}\left[h\;\left(\frac{1+\delta_{k}}{2}\right)-h\;\left(\frac{1+r_{k}\left(s_{k}\right)}{2}\right)\right],$$

so that the total von Neumann entropic cohesion in the aligned class is the sum of (54) over active pairs. Equation (54) shows explicitly how intrinsic imaginary coherence converts diagonal population uncertainty into spectral concentration.

### 5.3. Rényi-α Identity

The Rényi entropy of order α∈(0,1)∪(1,∞) of a density matrix ρ is

(55) $$S_{\alpha }\left(\rho \right)=\frac{1}{1-\alpha }\;\log\;\mathrm{tr}\left(\rho^{\alpha }\right),$$

which reduces to the von Neumann entropy −tr(ρlogρ) in the limit α→1 and to $-\log\mathrm{tr}(\rho^{2})$ at α=2. For an aligned-class state, the trace power $\mathrm{tr}(\rho^{\alpha })$ decomposes into a sum over the spectral structure described above,

(56) $$\mathrm{tr}\left(\rho^{\alpha }\right)=\sum_{k=1}^{\left\lfloor n/2\right\rfloor }\;[{\left(\lambda_{+}^{\left(k\right)}\right)}^{\alpha }+{\left(\lambda_{-}^{\left(k\right)}\right)}^{\alpha }]+\;\;\sum_{l\;\mathrm{unpaired}}\;\;a_{l}^{\;\alpha },$$

which is the Rényi-α generalization of the Frobenius identity (17). The first sum collects the contributions of the aligned Youla pairs and depends on both populations and Youla values; the second collects unpaired population contributions and is independent of the Youla values.

Substituting (49) into (56) yields, for each Youla pair, a contribution of the form

(57) $$F_{\alpha }\left(a_{i_{k}},a_{j_{k}},s_{k}\right)={\left(\frac{a_{i_{k}}+a_{j_{k}}+\Delta_{k}}{2}\right)}^{\;\alpha }\;\;+{\left(\frac{a_{i_{k}}+a_{j_{k}}-\Delta_{k}}{2}\right)}^{\;\alpha },$$

with $\Delta_{k}=\sqrt{{\left(a_{i_{k}}-a_{j_{k}}\right)}^{\;2}+4a_{i_{k}}a_{j_{k}}\;s_{k}^{\;2}}$, so that

(58) $$\mathrm{tr}\left(\rho^{\alpha }\right)=\sum_{k=1}^{\left\lfloor n/2\right\rfloor }F_{\alpha }\left(a_{i_{k}},a_{j_{k}},s_{k}\right)+\;\;\sum_{l\;\mathrm{unpaired}}\;\;a_{l}^{\;\alpha }.$$

This is the Rényi-α refinement of the hierarchical identity (16), valid in the aligned class for arbitrary α.

### 5.4. Interpolation Between Quadratic and Entropic Limits

At α=2, expanding $F_{2}$ gives

(59) $$F_{2}\left(u,v,s\right)=u^{2}+v^{2}+2uv\;s^{2},$$

and the identity (58) reduces, via $\mathrm{tr}\left(\rho^{2}\right)=\sum_{i}a_{i}^{\;2}+2\sum_{k}a_{i_{k}}a_{j_{k}}s_{k}^{\;2}$, to the scalar complementarity Formula (14) after the standard rearrangement that converts $\sum_{i}a_{i}^{\;2}$ into $1/n+\left(n-1\right)P_{p}^{\;2}/n$.

In the limit α→1, the function $F_{\alpha }\left(u,v,s\right)$ tends to the von Neumann contribution $-\lambda_{+}^{\left(k\right)}\log\lambda_{+}^{\left(k\right)}-\lambda_{-}^{\left(k\right)}\log\lambda_{-}^{\left(k\right)}$ of the pair, recovering the standard decomposition of the entropy over the spectrum. The Rényi-α family therefore provides a continuous interpolation between the algebraic identity of Theorem 1 and the entropic content of ρ.

At intermediate values of α, the identity (58) captures information-theoretic quantities of independent relevance. The collision entropy $S_{2}$ is included directly in (58); the limiting quantities $S_{\infty }=\lim_{\alpha \to \infty }S_{\alpha }$ and $S_{0}=\lim_{\alpha \to 0^{+}}S_{\alpha }$ quantify, respectively, optimal-guessing behavior and effective support size. Each of these quantities has, in the aligned class, an explicit expression in terms of the populations and Youla values through (58), making the IRB and the metaspin tensor framework directly applicable to information-theoretic problems beyond any specialized setting.

### 5.5. Entropic Cohesion

The intrinsic populations $\left\{a_{i}\right\}$ are the diagonal entries of $\rho_{O}$ in the IRB, while the eigenvalues $\left\{\lambda_{l}\right\}$ are the spectrum of ρ. The two sequences are related by the Schur majorization

(60) $$\left\{\lambda_{l}\right\}≻\left\{a_{i}\right\},$$

expressing that the eigenvalues majorize the diagonal entries, a standard consequence of the Hermitian structure of ρ together with the orthogonality of the IRB. Schur concavity of the Rényi entropies for all α>0 then implies the inequality

(61) $$S_{\alpha }\left(\rho \right)\leq S_{\alpha }\left(\left\{a_{i}\right\}\right),$$

with equality if and only if all Youla values $s_{m}$ vanish, in which case N=0 and the state is IRB-real. This condition is distinct from regularity in the discriminating sense used in the theory of nonregularity [15]. A state may have N≠0 and still have a vanishing imaginary part in its discriminating component. The gap between the two sides admits a natural interpretation as the *entropic cohesion* of the antisymmetric block,

(62) $$\Delta S_{\alpha }\left(\rho \right)=S_{\alpha }\left(\left\{a_{i}\right\}\right)-S_{\alpha }\left(\rho \right)\geq 0,$$

which quantifies the contribution of the intrinsic coherences to spectral concentration, equivalently to the reduction in spectral entropy relative to the intrinsic population distribution, at Rényi order α. At α=1, $\Delta S_{1}=S\left(A\right)-S\left(\rho \right)$ coincides with the relative entropy of coherence [4] evaluated after dephasing in the state-adapted IRB. The novelty claimed here is not a new definition of that standard entropy difference, but its intrinsic real-structure selection, its metaspin/Youla resolution, and its connection with the complementarity and spectral-sharpening identities. At α=2, the explicit form is

(63) $$\Delta S_{2}\left(\rho \right)=\log\left(\frac{\mathrm{tr}(\rho^{2})}{\sum_{i}a_{i}^{\;2}}\right)=\log\left(1+\frac{{∥N∥}_{F}^{\;2}}{\sum_{i}a_{i}^{\;2}}\right),$$

which is monotonically increasing in the cohesion squared and vanishes for IRB-real states. Since $0\leq S_{\alpha }\left(\rho \right)\leq \log n$ and $0\leq S_{\alpha }\left(\left\{a_{i}\right\}\right)\leq \log n$ for normalized *n*-level states, the gap satisfies the general bound $0\leq \Delta S_{\alpha }\leq \log n$ whenever the Rényi entropy is finite. The bound is not claimed to be sharp for all structural classes; for instance, pure states in the present intrinsic representation have additional rank restrictions on Reρ. The entropic cohesion $\Delta S_{\alpha }$ is an intrinsic quantifier relative to the specified real structure. It measures the entropy reduction associated with the antisymmetric content exposed in the IRB. It is not asserted here to be a monotone under the free operations of an operational resource theory. In the aligned class it admits an explicit expression in terms of the populations and Youla values through the spectrum (49).

## 6. The Dimensional Transition Revisited

The hierarchical identity (16) expresses the normalized purity of an aligned-class state as a sum of population contributions and pair-by-pair correlation contributions weighted by products of intrinsic populations. Within this framework, the dimensional transition (15) in the cohesion bound of the discriminating component acquires a transparent algebraic interpretation. The bound (15) is taken from Ref. [13], where the discriminating component and its extremal geometries are defined and analyzed; here, that result is reinterpreted in the language of the metaspin tensor and the hierarchical identity, without any additional assumption about the spectrum of the discriminating component beyond the properties established in that reference.

### 6.1. Discriminating Nonregularity and Scope of the Argument

The cohesion bound (15), namely $∥N\left(\rho_{d}\right){∥}_{F}\leq N_{n}$, concerns the antisymmetric block of the discriminating component $\rho_{d}$, not the full density matrix in arbitrary form. A state is nonregular, in the sense used here, when the discriminating component is not real. Equivalently, the decomposition of the state into its pure, discriminating, and maximally mixed contributions does not confine all imaginary content to the pure component; a genuinely imaginary contribution remains in $\rho_{d}$. This distinction is essential. The condition N≠0 for the total IRB representation $\rho_{O}=A+iN$ measures intrinsic imaginarity or cohesion of the state. It is not, by itself, the definition of nonregularity.

In the aligned configurations that saturate the bound, the discriminating contribution to the cohesion has the form

(64) $$∥N\left(\rho_{d}\right){∥}_{F}^{\;2}=2\sum_{k=1}^{K}a_{i_{k}}\;a_{j_{k}}\;s_{k}^{\;2},$$

where *K* is the number of active Youla pairs of the discriminant. Positivity implies $s_{k}\leq 1$, and saturation of a two-dimensional coherent sector corresponds to $s_{k}=1$. Equation (64) is therefore the algebraic object through which the known geometric bound (15) is read in the present framework.

### 6.2. Single-Pair Saturation in Dimension Three

In dimension three, an antisymmetric matrix has only one Youla value. Consequently, any discriminating imaginary content can occupy only one principal coherence pair. The extremal nonregular discriminating component found in Ref. [13] has intrinsic populations compatible with a single saturating pair, conventionally written as $\left(a_{2},a_{3}\right)$, with

(65) $$a_{2}=a_{3}=\frac{1}{4},\;s_{1}=1.$$

The correlation contribution is then

(66) $$N_{3}^{\;2}=2a_{2}a_{3}s_{1}^{2}=2\left(\frac{1}{4}\right)\left(\frac{1}{4}\right)=\frac{1}{8},\;N_{3}=\frac{1}{2\sqrt{2}}.$$

The value is thus controlled by a single nonzero term in the correlation sum of the hierarchical identity.

### 6.3. Doubly Coherent Saturation from Dimension Four Onward

From dimension four onward, two mutually orthogonal Youla pairs can be active simultaneously. The extremal discriminating geometry of Ref. [13] is then represented, in the intrinsic frame, by two saturating two-dimensional sectors. In the four-dimensional representative, one may take

(67) $$a_{1}=a_{2}=a_{3}=a_{4}=\frac{1}{4},\;s_{1}=s_{2}=1,$$

with active pairs (1,2) and (3,4). The correlation contribution becomes

(68) $$N_{4}^{\;2}=2\left(a_{1}a_{2}+a_{3}a_{4}\right)=2\left(\frac{1}{16}+\frac{1}{16}\right)=\frac{1}{4},\;N_{4}=\frac{1}{2}.$$

The increase from $N_{3}$ to $N_{4}$ is therefore the square root of the increase from one saturating contribution to two equal saturating contributions in the pairwise correlation sum.

For n>4, the same extremal two-sector construction embeds into the larger Hilbert space by adding passive intrinsic axes that carry no contribution to the discriminating cohesion. Additional ambient dimensions make further Youla planes available, but the established bound shows that they do not increase the maximum cohesion of the discriminating component. In the hierarchical language, this means that the two-sector saturating pattern is already optimal for the discriminating geometry; extra pairs would either be inactive or redistribute the available population weight rather than raising the value of (64).

### 6.4. The Plateau as a Metaspin Saturation Pattern

The dimensional transition

(69) $$N_{3}=\frac{1}{2\sqrt{2}}\;⟶\;N_{n\geq 4}=\frac{1}{2}$$

is therefore a transition in the metaspin saturation pattern of the discriminating component, from one active saturating Youla pair in dimension three to two active saturating Youla pairs in dimensions four and higher. The plateau for n≥4 follows from the fact that the doubly coherent two-sector pattern can be embedded in all higher dimensions without changing its active intrinsic support. This is the algebraic counterpart of the conjugate-orthogonal two-sector construction of Ref. [13], and it explains the saturation pattern underlying the discontinuity summarized by the scalar descriptor $P_{c}$.

## 7. Examples

This section illustrates the hierarchical identity (16) and the structural results of Section 4, Section 5 and Section 6 on representative configurations in dimensions three, four, and five. The examples are chosen to expose distinct aspects of the framework. They include a rank-two three-dimensional state with one active intrinsic coherence pair, an explicit two-qubit depolarized Bell state with complete IRB construction, a family of four-dimensional states that share a common scalar normalized purity while differing in the distribution of correlation across Youla pairs, and a five-dimensional state that embeds the four-dimensional doubly coherent configuration and demonstrates the dimensional plateau of $N_{n}$.

### 7.1. Three-Dimensional Rank-Two State with One Active Coherence Pair

Consider the density matrix

(70) $$\rho =\left(\begin{array}{ccc} 1/2 & 1/6+i\sqrt{2}/6 & 0\\ 1/6-i\sqrt{2}/6 & 1/2 & 0\\ 0 & 0 & 0 \end{array}\right),$$

which has eigenvalues $\lambda_{1,2}=1/2\pm 1/\left(2\sqrt{3}\right)$ and $\lambda_{3}=0$, hence rank two. In the IRB of ρ, obtained by diagonalizing the real part, the intrinsic representation becomes

(71) $$\rho_{O}=\left(\begin{array}{ccc} 2/3 & 0 & 0\\ 0 & 1/3 & 0\\ 0 & 0 & 0 \end{array}\right)+i\left(\begin{array}{ccc} 0 & \sqrt{2}/6 & 0\\ -\sqrt{2}/6 & 0 & 0\\ 0 & 0 & 0 \end{array}\right),$$

with intrinsic populations (2/3,1/3,0) and a single nonzero coherence $N_{12}=\sqrt{2}/6$. The state is in the aligned class with one Youla pair (1,2) and Youla value

(72) $$s_{1}=\frac{N_{12}}{\sqrt{a_{1}a_{2}}}=\frac{\sqrt{2}/6}{\sqrt{2}/3}=\frac{1}{2},$$

halfway to saturation. The population indices are

(73) $$M_{1}=a_{1}-a_{2}=\frac{1}{3},\;M_{2}=a_{1}+a_{2}-2a_{3}=1.$$

Application of the hierarchical identity (16) gives

(74) $$P_{3D}^{\;2}=\frac{3}{2}\left[\frac{{\left(1/3\right)}^{2}}{2}+\frac{1^{2}}{6}+2\cdot \frac{2}{9}\cdot \frac{1}{4}\right]=\frac{1}{2},$$

in agreement with the direct evaluation $P_{3D}^{\;2}=\left(3\mathrm{tr}\left(\rho^{2}\right)-1\right)/2=1/2$. The cohesion of the total antisymmetric block is

(75) $${∥N∥}_{F}=\sqrt{2N_{12}^{\;2}}=\frac{1}{3}.$$

This example also illustrates the distinction between intrinsic cohesion and nonregularity. Although N≠0, the discriminating component of this state is real, i.e., $N(\rho_{d})=0$; hence, the state is regular in the discriminating sense. Its nonzero *N* measures IRB imaginarity of the full state, not nonregularity of a nonreal discriminating component.

### 7.2. Four-Dimensional Depolarized Bell State as a Two-Qubit Example

A standard two-qubit example makes the construction explicit in a familiar setting. Let

(76) $$|\Phi_{i}\rangle =\frac{|00\rangle +i|11\rangle }{\sqrt{2}},\;0\leq q\leq 1,$$

and consider the depolarized Bell state

(77) $$\rho_{B}\left(q\right)=\frac{1-q}{4}I_{4}+q|\Phi_{i}\rangle \langle \Phi_{i}|=\left(\begin{array}{cccc} \frac{1+q}{4} & 0 & 0 & -\frac{iq}{2}\\ 0 & \frac{1-q}{4} & 0 & 0\\ 0 & 0 & \frac{1-q}{4} & 0\\ \frac{iq}{2} & 0 & 0 & \frac{1+q}{4} \end{array}\right)$$

in the computational ordering (|00〉,|01〉,|10〉,|11〉). The state is locally phase-equivalent to the usual depolarized $|\Phi^{+}\rangle$ Bell state; the phase has been chosen only to make the imaginary coherence sector visible in the fixed real structure.

The real part of (77) is already diagonal, but the ordered IRB is obtained by the permutation (|00〉,|11〉,|01〉,|10〉). In that ordered IRB,

(78) $$A=\mathrm{diag}\;\left(\frac{1+q}{4},\frac{1+q}{4},\frac{1-q}{4},\frac{1-q}{4}\right),\;N_{12}=-\frac{q}{2},$$

with all other independent entries of *N* equal to zero. Hence, the state lies in the aligned class with one active pair (1,2) and

(79) $$s_{1}=\frac{|N_{12}|}{\sqrt{a_{1}a_{2}}}=\frac{2q}{1+q},\;s_{2}=0.$$

The intrinsic population-asymmetry indices are

(80) $$M_{1}=0,\;M_{2}=q,\;M_{3}=q.$$

The hierarchical identity gives

(81) $$P_{4D}^{\;2}=\frac{4}{3}\left[\frac{q^{2}}{6}+\frac{q^{2}}{12}+2{\left(\frac{1+q}{4}\right)}^{2}{\left(\frac{2q}{1+q}\right)}^{2}\right]=q^{2}.$$

The direct calculation from the ordinary purity gives the same result, since the spectrum is ((1+3q)/4,(1−q)/4,(1−q)/4,(1−q)/4) and therefore

(82) $$\mathrm{tr}\left(\rho_{B}{\left(q\right)}^{2}\right)=\frac{1}{4}+\frac{3q^{2}}{4},\;P_{4D}^{\;2}=\frac{4\mathrm{tr}(\rho_{B}{\left(q\right)}^{2})-1}{3}=q^{2}.$$

Thus, the usual purity compresses the state to a single scalar $q^{2}$, whereas the hierarchical representation identifies that one third of the normalized purity comes from the population hierarchy and two thirds from the intrinsic imaginary coherence of the Bell pair. This example illustrates the structural readability of the IRB construction in a conventional two-qubit state.

### 7.3. Four-Dimensional Doubly Coherent Versus Single-Pair-Dominated Family

A one-parameter family of four-dimensional density matrices that interpolates between a single-pair-dominated configuration and the doubly coherent perfectly nonregular discriminating configuration is constructed by

(83) $$\rho_{d}\left(\chi \right)=\frac{1}{2}\;|\psi_{\chi }\rangle \langle \psi_{\chi }|+\frac{1}{2}\;|\varphi_{\pi /4}\rangle \langle \varphi_{\pi /4}|,$$

with $|\psi_{\chi }\rangle =\cos\chi \;|0\rangle +i\sin\chi \;|1\rangle$ and $|\varphi_{\pi /4}\rangle =(|2\rangle +i|3\rangle )/\sqrt{2}$, where χ∈[0,π/4] parametrizes the mixing angle of the first contribution. In the fixed basis ordering displayed in (83), the two coherent sectors are (0,1) and (2,3), and the corresponding diagonal entries are

(84) $$\left(\frac{\cos^{2}\chi }{2},\frac{\sin^{2}\chi }{2},\frac{1}{4},\frac{1}{4}\right).$$

For χ≠π/4, these entries are not necessarily in nonincreasing order. If the IRB ordering convention $a_{1}\geq a_{2}\geq a_{3}\geq a_{4}$ is enforced, the same permutation must be applied simultaneously to the coherent pairs. Equivalently, one may keep the fixed basis ordering for this example and evaluate the pair contribution directly; the scalar purity and cohesion are, of course, invariant under the simultaneous relabeling.

Both active sectors are internally saturated whenever their two populations are nonzero. The cohesion is therefore

(85) $${∥N∥}_{F}^{\;2}=2\left[\frac{\cos^{2}\chi }{2}\frac{\sin^{2}\chi }{2}+\frac{1}{4}\frac{1}{4}\right]=\frac{\sin^{2}\left(2\chi \right)}{8}+\frac{1}{8}.$$

It interpolates monotonically between ${∥N∥}_{F}^{\;2}=1/8$ at χ=0, where only the second coherent sector contributes because the first has zero population product, and ${∥N∥}_{F}^{\;2}=1/4$ at χ=π/4, where the doubly coherent perfectly nonregular discriminating configuration realizes $N_{4}=1/2$.

**Figure 1:**
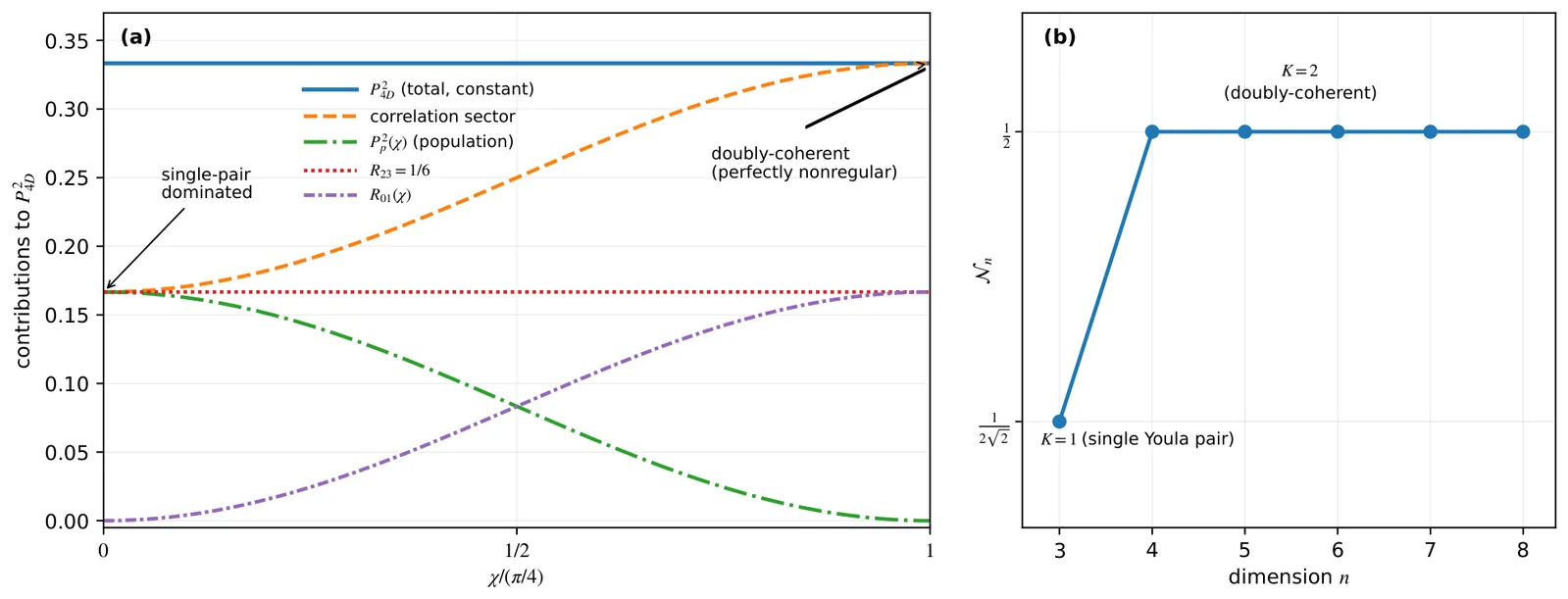
Two manifestations of the hierarchical structure of the intrinsic representation. Panel (**a**) shows the decomposition of the constant normalized purity $P_{4D}^{\;2}=1/3$ along the four-dimensional family $\rho_{d}\left(\chi \right)$ of (83). The scalar identity displays the total population contribution $P_{p}^{\;2}$ and total correlation contribution, whereas the hierarchical identity further resolves the correlation sector into the constant (2,3)-pair contribution and the continuously activated (0,1)-pair contribution. The endpoint χ=0 is single-pair-dominated and χ=π/4 is the doubly coherent perfectly nonregular discriminating configuration; at the latter degenerate-Youla endpoint, the pair-resolved display refers to the aligned representative selected continuously by the family (83). Panel (**b**) shows the dimensional transition in the cohesion bound $N_{n}$ of the discriminating component, $N_{3}=1/\left(2\sqrt{2}\right)$ and $N_{n\geq 4}=1/2$. The transition by a factor of $\sqrt{2}$ corresponds algebraically to the increase from one to two simultaneously saturating Youla pairs of the discriminant’s metaspin tensor (Section 6).

The spectrum of $\rho_{d}\left(\chi \right)$ is independent of χ throughout the family, with eigenvalues (1/2,1/2,0,0). The scalar normalized purity therefore remains constant, $P_{4D}^{\;2}=1/3$, across the family. By contrast, the hierarchical identity (16) resolves the family by tracking how the constant $P_{4D}^{\;2}$ is partitioned between the population sector and the correlation sector. At χ=0, the first coherent sector is rank-deficient and makes no correlation contribution, whereas at χ=π/4, both saturated sectors contribute equally. The scalar formula already records the transfer between the total population and total correlation sectors. The hierarchical identity refines this information by separating the two pairwise correlation contributions. The (2,3) sector contributes a constant term, whereas the (0,1) sector is activated continuously as χ increases (Figure 1a). States with the same global normalized purity can therefore differ in the algebraic distribution of their cohesion among intrinsic pair sectors.

For 0<χ≤π/4, both active two-level sectors have saturating Youla values $s_{1}=s_{2}=1$, and the indices of correlation asymmetry are therefore $C_{1}=0$ and $C_{2}=2$. At the endpoint χ=0, the first sector is rank-deficient. Its population product vanishes and it contributes no cohesion, so the hierarchy must be read with the pseudoinverse convention for zero intrinsic populations. The endpoint is thus a limiting single-pair-dominated configuration, whereas χ=π/4 is the genuinely doubly coherent perfectly nonregular discriminating configuration. The scalar identity records the total transfer between population and correlation sectors, while the hierarchical identity additionally records which intrinsic pair supplies each part of the correlation contribution.

### 7.4. Five-Dimensional Rank-Four Embedded Doubly Coherent

The dimensional plateau of the cohesion bound $N_{n}=1/2$ for n≥4 is exhibited by the five-dimensional state

(86) $$\rho_{d}^{\max,5}=\frac{1}{2}\;|\psi_{\pi /4}\rangle \langle \psi_{\pi /4}|+\frac{1}{2}\;|\varphi_{\pi /4}\rangle \langle \varphi_{\pi /4}|,$$

identical in structure to the doubly coherent state of Section 7.3 but embedded in five-dimensional Hilbert space, with the fifth axis carrying zero population. The state is in the aligned class with two Youla pairs (1,2) and (3,4), both saturating ($s_{1}=s_{2}=1$), and a fifth unpaired axis with $a_{5}=0$. The cohesion is ${∥N∥}_{F}=1/2=N_{5}$, in agreement with the dimensional plateau, and the hierarchical identity gives

(87) $$P_{5D}^{\;2}\left(\rho_{d}^{\max,5}\right)=\frac{5}{4}[\sum_{k=1}^{4}\frac{M_{k}^{\;2}}{k(k+1)}+2\left(a_{1}a_{2}+a_{3}a_{4}\right)],$$

with the population indices $\left(M_{1},M_{2},M_{3},M_{4}\right)=\left(0,0,0,1\right)$ corresponding to the rank-four structure of the embedded state. The population sum reduces to the single nonzero contribution $M_{4}^{\;2}/20=1/20$, and the correlation sum to 1/4, giving $P_{5D}^{\;2}=\left(5/4\right)\left(1/20+1/4\right)=\left(5/4\right)\left(6/20\right)=3/8$. The direct calculation from $\mathrm{tr}(\rho^{2})=1/2$ gives the same value, $P_{5D}^{\;2}=\left(5\cdot 1/2-1\right)/4=3/8$. The hierarchical identity therefore matches the direct evaluation, and the algebraic role of the dimensional embedding is made manifest. The additional axis with zero population contributes only through the index $M_{n-1}$ of population asymmetry, which records the rank deficiency of the state, and does not affect the correlation sector. The bound $N_{5}=1/2$ is saturated by the same doubly coherent configuration that saturates $N_{4}$, with the extra dimension passive at the correlation level.

## 8. Discussion

The results above give a representation-intrinsic hierarchy for finite-dimensional density matrices in which ordered populations, imaginary coherences, spectral sharpening, and entropy are linked by explicit algebraic identities. The framework is not a replacement for unitary spectral analysis; rather, it complements it by identifying an intrinsic real frame in which the diagonal population sector and the antisymmetric imaginary sector can be compared level by level. Four points are worth emphasizing.

First, the aligned-class identity is exact and pair-resolved. It refines the scalar purity decomposition by retaining the distribution of the imaginary contribution across intrinsic two-level sectors. This is useful because scalar purity can remain unchanged while the population and coherence contributions are redistributed across different intrinsic sectors. The bilinear form of Theorem 3 shows how the same information is encoded away from alignment. The price of arbitrary orientation is not a loss of exactness but the appearance of population-weighted Plücker overlaps between Youla two-planes.

Second, Theorem 5 gives the central information-theoretic consequence of the construction. In the aligned class, intrinsic coherence is a spectral-sharpening mechanism. Increasing the Youla values at fixed populations and fixed pairing makes the spectrum more majorized and therefore lowers every Rényi entropy. The binary expression (54) gives an explicit von Neumann entropy loss for each intrinsic pair. This gives the metaspin tensor a direct information-theoretic interpretation. In the aligned class, its Youla values quantify how much diagonal population uncertainty is converted into spectral concentration.

Third, the fixed-population optimization theorem, Theorem 2, shows that the extremal aligned configuration is not arbitrary. For ordered populations, maximum aligned cohesion is obtained by saturating adjacent population pairs. This places the aligned class on a variational footing and suggests a natural hierarchy of extremal states obtained by ordering the populations, pairing adjacent populations, and saturating the allowed Youla values. The same mechanism underlies the algebraic reinterpretation of the discriminating-component transition from $N_{3}$ to $N_{n\geq 4}$.

Fourth, the distinction between IRB imaginarity and discriminating nonregularity is essential. A state with N≠0 in its intrinsic representation has nonzero imaginary coherence relative to the specified real structure; whether this constitutes a nonzero operational resource of imaginarity depends on the chosen free operations. It is nonregular only when the discriminating component of the discriminating decomposition is itself nonreal. Keeping these notions separate makes the framework compatible both with resource-theoretic language and with the geometric theory of nonregularity.

### 8.1. Relevance for Quantum Information and Resource Theories

The antisymmetric block *N* is the part of the intrinsic representation that distinguishes ρ from a real density matrix in the IRB. This suggests an interface with resource theories of imaginarity [5,6], in which a real structure and a class of free operations are specified independently. Relative to the real structure fixed here, the Frobenius norm ${∥N∥}_{F}$, the Youla values $s_{k}$, and the indices of correlation asymmetry $C_{k}$ provide a hierarchy of intrinsic imaginary-content descriptors. In the aligned class, these descriptors have explicit consequences for purity, spectrum, and entropy. In arbitrary orientation, their effect is mediated by the bilinear matrix *G*.

The relation to coherence resource theory [4,7,8] is complementary. Standard coherence measures depend on a chosen reference basis and are studied together with a prescribed class of free operations. Here, the reference basis is not externally imposed but obtained intrinsically from the real part of the state, after the real structure has been specified. Consequently, the present descriptors should not be read as standard coherence monotones. They are state-adapted structural descriptors that explain how diagonal population uncertainty and imaginary coherence combine to determine spectral and entropic quantities. This is also why the discussion is formulated cautiously with respect to the general resource-theoretic framework [9].

The connection with imaginarity is similarly precise but limited in scope. The IRB defines a state-adapted real frame, whereas an operational resource theory normally fixes the free real structure and the free operations independently of the state. The equality $\Delta S_{1}=S\left(A\right)-S\left(\rho \right)$ with the relative entropy of coherence after dephasing in the IRB identifies the entropic quantity involved, but the adaptive choice of IRB changes the operational question. A natural next step is to characterize the class of operations that preserve or decrease the alignment defect and the correlation-asymmetry hierarchy. Such a characterization would be a step toward a resource theory of intrinsic imaginary coherence, with the IRB playing the role of an adaptively selected free frame.

### 8.2. State-Space Geometry

The bilinear form (30) gives a geometric interpretation of the departure from alignment. Each Youla two-plane defines a point of the Grassmannian Gr(2,n) through its Plücker coordinates, and the population vector induces a weighted metric on these coordinates. The matrix *G* is therefore a population-weighted Gram matrix of the Youla two-planes. For an aligned state evaluated in an aligned IRB/Youla representative, the Gram structure is diagonal; nonaligned states may also lie on that diagonal locus when their Youla planes are orthogonal in the population-weighted metric. Away from that diagonal locus, off-diagonal overlaps encode how different coherence planes interfere when projected onto the population-weighted intrinsic frame.

This geometric reading suggests several mathematically precise problems. One is to determine the global maximum of ${∥N∥}_{F}^{2}$ for fixed populations and fixed Youla values over all orientations $Q_{M}$. Another is to characterize the image of the map $\left(a,Q_{M}\right)\mapsto G\left(a,Q_{M}\right)$ subject to Plücker constraints. A third is to identify whether the adjacent-pairing maximum of Theorem 2 remains globally optimal beyond the aligned class under suitable spectral constraints. These questions are deliberately left open because they require optimization over Grassmannian orbits rather than the elementary matching argument used here.

### 8.3. Open Questions

Three directions appear especially promising. First, the residual-invariant alignment defect $\Lambda_{\mathrm{IRB}}\left(\rho \right)$ is monotonic under the partial-dephasing family considered in Proposition 2. The maximal class of completely positive trace-preserving maps under which $\Lambda_{\mathrm{IRB}}$ is monotonic remains to be determined.

Second, the eigenstate sum rule of Theorem 4 constrains the antisymmetric blocks of all orthonormal pure-state decompositions. In dimension three, it reduces to a familiar coplanarity-type constraint; in higher dimensions, it defines an algebraic variety of antisymmetric matrix tuples. A complete description of this variety would provide a higher-dimensional eigenstate geometry adapted to the IRB.

Third, the entropic cohesion $\Delta S_{\alpha }$ and the correlation-asymmetry hierarchy $C_{k}$ suggest a multi-level resource structure. The present article proves monotonic spectral sharpening in the aligned class, but a full operational interpretation of the individual $C_{k}$ under restricted free operations remains open. Establishing such an interpretation could identify which combinations of the hierarchical descriptors behave as resource monotones with direct information-theoretic meaning.

The main conclusion is that intrinsic population–coherence complementarity is not only a quadratic purity identity. It is a hierarchy of algebraic, geometric, and entropic relations that provides a structured language for finite-dimensional quantum-state analysis.

## Data Availability

No new data were created or analyzed in this study. Data sharing is not applicable to this article. This article is theoretical and did not generate or analyze datasets.

## Appendix A. Explicit Treatment of the Bilinear Form in Dimension Four via *SO*(4) ≅ (*SU*(2) × *SU*(2))/Z_2_

The bilinear form of Theorem 3 admits a particularly transparent treatment in dimension four, where the isomorphism $SO\left(4\right)≅\left(SU\left(2\right)\times SU\left(2\right)\right)/Z_{2}$ decomposes the action of orthogonal congruences on 2-forms into independent self-dual and anti-self-dual rotations. The purpose of this appendix is to give an expression for ${∥N∥}_{F}^{\;2}$ in which the Youla amplitudes are separated from the orientation of the corresponding two-planes. Throughout the appendix, unit orientation vectors are denoted with hats; all amplitude dependence is carried explicitly by $s_{1},s_{2}$ or by $A_{\pm }=s_{1}\pm s_{2}$. After fixing the overall handedness of a Youla representative, orientation-preserving variations are described by SO(4); reflections alter only the handedness convention and do not change the cohesion expressions below.

### Appendix A.1. Self-Dual and Anti-Self-Dual Decomposition

The space $\Lambda^{2}\left(R^{4}\right)$ of 2-forms is six-dimensional and decomposes under the Hodge star operator ⋆ into the three-dimensional eigenspaces $\Lambda_{\pm }^{2}\left(R^{4}\right)$ of ⋆ at eigenvalues ±1. An orthonormal basis of $\Lambda_{\pm }^{2}$ adapted to the standard basis ${\left\{e_{i}\right\}}_{i=1}^{4}$ of $R^{4}$ is

(A1) $$\omega_{1}^{\pm }=\frac{1}{\sqrt{2}}\left(e_{1}\wedge e_{2}\pm e_{3}\wedge e_{4}\right),\;\omega_{2}^{\pm }=\frac{1}{\sqrt{2}}\left(e_{1}\wedge e_{3}\mp e_{2}\wedge e_{4}\right),\;\omega_{3}^{\pm }=\frac{1}{\sqrt{2}}\left(e_{1}\wedge e_{4}\pm e_{2}\wedge e_{3}\right).$$

The SO(4) action on $\Lambda^{2}$ preserves $\Lambda_{\pm }^{2}$, with the restriction to $\Lambda_{+}^{2}$ corresponding to the $SU{\left(2\right)}_{+}$ factor and the restriction to $\Lambda_{-}^{2}$ to the $SU{\left(2\right)}_{-}$ factor of $SO\left(4\right)≅\left(SU\left(2\right)\times SU\left(2\right)\right)/Z_{2}$.

### Appendix A.2. Unit Orientation Vectors and Youla Amplitudes

Let M be a real antisymmetric matrix in dimension four with Youla values $s_{1}\geq s_{2}\geq 0$. Let $\pi^{\left(1\right)}$ and $\pi^{\left(2\right)}$ be the two decomposable unit 2-forms associated with the two orthogonal Youla planes. Modulo the residual SO(2)×SO(2) stabilizer of the two-plane decomposition, a pair of unit vectors

(A2) $${\hat{v}}_{+},{\hat{v}}_{-}\in S^{2}\subset R^{3}$$

parametrizes the self-dual and anti-self-dual orientation of the first plane. Its Plücker coordinates are

(A3) $$\begin{array}{cccc} \pi_{12}^{\left(1\right)} & =\frac{1}{2}\left({\hat{v}}_{+,1}+{\hat{v}}_{-,1}\right), & \;\pi_{34}^{\left(1\right)} & =\frac{1}{2}\left({\hat{v}}_{+,1}-{\hat{v}}_{-,1}\right),\\ \pi_{13}^{\left(1\right)} & =\frac{1}{2}\left({\hat{v}}_{+,2}+{\hat{v}}_{-,2}\right), & \;\pi_{24}^{\left(1\right)} & =\frac{1}{2}\left(-{\hat{v}}_{+,2}+{\hat{v}}_{-,2}\right),\\ \pi_{14}^{\left(1\right)} & =\frac{1}{2}\left({\hat{v}}_{+,3}+{\hat{v}}_{-,3}\right), & \;\pi_{23}^{\left(1\right)} & =\frac{1}{2}\left({\hat{v}}_{+,3}-{\hat{v}}_{-,3}\right). \end{array}$$

The second plane is the orthogonal complement and is obtained by reversing the anti-self-dual orientation,

(A4) $$\pi_{ij}^{\left(2\right)}\left({\hat{v}}_{+},{\hat{v}}_{-}\right)=\pi_{ij}^{\left(1\right)}\left({\hat{v}}_{+},-{\hat{v}}_{-}\right).$$

The metaspin tensor, viewed as a 2-form, is

(A5) $$M=s_{1}\pi^{\left(1\right)}+s_{2}\pi^{\left(2\right)}.$$

It is useful to introduce the self-dual and anti-self-dual amplitudes

(A6) $$A_{+}=s_{1}+s_{2},\;A_{-}=s_{1}-s_{2}.$$

Then, the SD and ASD components of M are proportional to $A_{+}{\hat{v}}_{+}$ and $A_{-}{\hat{v}}_{-}$, respectively. This separation avoids any double counting of amplitudes. The unit directions ${\hat{v}}_{\pm }$ carry orientation only, while $A_{\pm }$ carry the invariant magnitudes.

### Appendix A.3. Population-Weighted Biases

The bilinear form *G* of Theorem 3 depends on the populations through the six products $a_{i}a_{j}$ for i<j. These products partition into the following three complementary 2-pair axes.

(A7) $$\begin{array}{cccc} P_{1,+} & =a_{1}a_{2}, & \;P_{1,-} & =a_{3}a_{4},\\ P_{2,+} & =a_{1}a_{3}, & \;P_{2,-} & =a_{2}a_{4},\\ P_{3,+} & =a_{1}a_{4}, & \;P_{3,-} & =a_{2}a_{3}. \end{array}$$

The symmetric and antisymmetric combinations on each axis are

(A8) $$\sigma_{a}=\frac{1}{2}\left(P_{a,+}+P_{a,-}\right),\;\delta_{a}=\frac{1}{2}\left(P_{a,+}-P_{a,-}\right),$$

with $\sigma_{a}\geq \left|\delta_{a}\right|\geq 0$. The vector σ records the population weights that are blind to exchange of complementary pairs, whereas δ records the bias between each pair and its complement.

### Appendix A.4. Explicit Form of the Bilinear Form

Substitution of (A3) and (A4) into the definition (31) of *G* yields

(A9) $$\begin{array}{cc} G_{11} & ={\hat{v}}_{+}^{T}\mathrm{diag}\left(\sigma \right){\hat{v}}_{+}+{\hat{v}}_{-}^{T}\mathrm{diag}\left(\sigma \right){\hat{v}}_{-}+2\;{\hat{v}}_{+}^{T}\mathrm{diag}\left(\delta \right){\hat{v}}_{-},\\ G_{22} & ={\hat{v}}_{+}^{T}\mathrm{diag}\left(\sigma \right){\hat{v}}_{+}+{\hat{v}}_{-}^{T}\mathrm{diag}\left(\sigma \right){\hat{v}}_{-}-2\;{\hat{v}}_{+}^{T}\mathrm{diag}\left(\delta \right){\hat{v}}_{-},\\ G_{12} & ={\hat{v}}_{+}^{T}\mathrm{diag}\left(\sigma \right){\hat{v}}_{+}-{\hat{v}}_{-}^{T}\mathrm{diag}\left(\sigma \right){\hat{v}}_{-}. \end{array}$$

Equivalently, using the amplitudes $A_{\pm }=s_{1}\pm s_{2}$, the cohesion takes the separated form

(A10) $${∥N∥}_{F}^{\;2}=\sigma_{+}\;A_{+}^{\;2}+\sigma_{-}\;A_{-}^{\;2}+2\;\delta_{\;\mathrm{pair}}\;A_{+}A_{-},$$

where

(A11) $$\sigma_{+}={\hat{v}}_{+}^{T}\mathrm{diag}\left(\sigma \right){\hat{v}}_{+},\;\sigma_{-}={\hat{v}}_{-}^{T}\mathrm{diag}\left(\sigma \right){\hat{v}}_{-},\;\delta_{\;\mathrm{pair}}={\hat{v}}_{+}^{T}\mathrm{diag}\left(\delta \right){\hat{v}}_{-}.$$

The diagonal terms in (A10) are the SD and ASD amplitude contributions weighted by the corresponding population biases, while the mixed term measures the projection of the population-pair asymmetry onto the relative SD/ASD orientation.

### Appendix A.5. Aligned Class and Special Configurations

The aligned class corresponds to

(A12) $${\hat{v}}_{+}={\hat{v}}_{-}=e_{a},\;a\in \left\{1,2,3\right\},$$

where the three choices of *a* select the pair structures {(1,2),(3,4)}, {(1,3),(2,4)}, and {(1,4),(2,3)}. In this case the bilinear form reduces to

(A13) $$G_{11}=2P_{a,+},\;G_{22}=2P_{a,-},\;G_{12}=0,$$

in agreement with the aligned limit of Theorem 3. Notice that it is the unit directions ${\hat{v}}_{\pm }$, not amplitude-carrying vectors, that are pinned to the discrete aligned orientations.

Two further configurations are useful. If all four intrinsic populations are equal, then $\delta_{a}=0$ for all *a* and $\sigma_{a}$ is independent of *a*; the bilinear form becomes a scalar multiple of the identity and ${∥N∥}_{F}^{\;2}$ depends only on the Youla spectrum, not on its orientation. If ${\hat{v}}_{+}=\pm {\hat{v}}_{-}$ without being aligned with an intrinsic axis, the mixed term is controlled by a single common orientation; the off-diagonal entry $G_{12}$ may vanish even though the state is not aligned in the stronger sense of Section 3.9.

### Appendix A.6. Departure from Alignment in Dimension Four

The off-diagonal entry $G_{12}$ vanishes if and only if

(A14) $${\hat{v}}_{+}^{T}\mathrm{diag}\left(\sigma \right){\hat{v}}_{+}={\hat{v}}_{-}^{T}\mathrm{diag}\left(\sigma \right){\hat{v}}_{-}.$$

This condition characterizes the locus where the population-weighted bilinear form is diagonal in the Youla basis. It is weaker than alignment of the Youla planes with pairs of IRB axes.

A dimensionless scalar measuring this weaker diagonality obstruction is

(A15) $$\Lambda_{G}^{\left(4\right)}\left(\rho \right)=\frac{G_{12}^{\;2}}{G_{11}\;G_{22}}=\frac{{\left(\sigma_{+}-\sigma_{-}\right)}^{2}}{{\left(\sigma_{+}+\sigma_{-}\right)}^{2}-4\;\delta_{\;\mathrm{pair}}^{2}},$$

whenever $G_{11}G_{22}>0$. It vanishes on the continuous locus where *G* is diagonal in the Youla basis. Within its domain, it reaches the boundary value one on the rank-deficient boundary detG=0; rank-deficient cases with $G_{11}G_{22}=0$ lie outside the domain of the normalized expression. When $\Lambda_{\mathrm{IRB}}\left(\rho \right)=0$, an aligned IRB/Youla representative can be selected; in that representative, whenever $\Lambda_{G}^{\left(4\right)}$ is defined, one has $\Lambda_{G}^{\left(4\right)}=0$. The converse need not hold because a diagonal *G* does not force support alignment. If degenerate intrinsic populations permit different residual representatives, no representative-independent implication for $\Lambda_{G}^{\left(4\right)}$ is asserted beyond this existence statement.
